# Fungi associated with dead branches of *Magnolia grandiflora*: A case study from Qujing, China

**DOI:** 10.3389/fmicb.2022.954680

**Published:** 2022-08-04

**Authors:** Nalin N. Wijayawardene, Dong-Qin Dai, Mei-Ling Zhu, Dhanushka N. Wanasinghe, Jaturong Kumla, Gui-Qing Zhang, Ting-Ting Zhang, Li-Su Han, Saowaluck Tibpromma, Huan-Huan Chen

**Affiliations:** ^1^Center for Yunnan Plateau Biological Resources Protection and Utilization, Yunnan Engineering Research Center of Fruit Wine, College of Biological Resource and Food Engineering, Qujing Normal University, Qujing, China; ^2^Section of Genetics, Institute for Research and Development in Health and Social Care, Battaramulla, Sri Lanka; ^3^Centre for Mountain Futures, Kunming Institute of Botany, Chinese Academy of Sciences, Kunming, China; ^4^Department of Economic Plants and Biotechnology, Yunnan Key Laboratory for Wild Plant Resources, Kunming Institute of Botany, Chinese Academy of Sciences, Kunming, China; ^5^Research Centre of Microbial Diversity and Sustainable Utilization, Chiang Mai University, Chiang Mai, Thailand

**Keywords:** *Ascomycota*, *Botryosphaeriales*, diversity, phylogeny, *Pleosporales*, taxonomy

## Abstract

As a result of an ongoing survey of microfungi associated with garden and ornamental plants in Qijing, Yunnan, China, several saprobic fungal taxa were isolated from *Magnolia grandiflora*. Both morphological and combined SSU, LSU, ITS, *tef1*, and *rpb2* locus phylogenetic analyses (maximum-likelihood and Bayesian analyses) were carried out to identify the fungal taxa. Three new species are introduced in *Pleosporales, viz*., *Lonicericola qujingensis* (*Parabambusicolaceae*), *Phragmocamarosporium magnoliae*, and *Periacma qujingensis* (*Lentitheciaceae*). *Botryosphaeria dothidea, Diplodia mutila*, and *Diplodia seriata* (in *Botryosphaeriaceae*) are reported from *Magnolia grandiflora* for the first time in China. *Angustimassarina populi* (*Amorosiaceae*) is reported for the first time on *M. grandiflora* from China, and this is the first report of a member of this genus outside Europe. *Shearia formosa* is also reported for the first time on *M. grandiflora* from China.

## Introduction

Discovering missing taxa in the fungal tree (or in Kingdom *Fungi*) is one of the popular topics among taxonomists. Recent species estimation studies have predicted that tropical regions harbor higher fungal diversity than previously expected (Hawksworth and Lücking, 2017; Hyde et al., 2020). Several studies (which were based on DNA sequence analyses) described numerous fungal species during the last decade from tropical countries such as, Thailand and India (Chaiwan et al., 2020; Calabon et al., 2021; Rajeshkumar et al., 2021). A large number of fungal species have also been described from subtropical China, especially in Yunnan and Guizhou provinces (Luo et al., 2018; Lu et al., 2021; Ren et al., 2021; Wang et al., 2021; Wijayawardene et al., 2021). These studies mentioned that a large number of fungal species are waiting to be discovered in Southwestern China, including the Guizhou and Yunnan Provinces.

*Magnolia grandiflora* (Southern magnolia) is an evergreen tree that is widely used as an ornamental plant in landscaping (Liu et al., 2019) and a fungal-rich host plant genus and is reported with over 1,000 records of taxa in Farr and Rossman (2022). Seventy-two (72) records have been mainly listed from different substrates of *Magnolia alba, M. delavayi, M. denudate*, and *M. grandiflora* from China (Farr and Rossman, 2022). Recently, Wanasinghe et al. (2020) studied fungi associated with *Magnolia* species in the Kunming Botanical Garden and predicted rich fungal diversity.

In this study, we collected ascomycetous fungi (both sexual and asexual morphs) that occur on different substrates of *M. grandiflora* from Qujing Normal University garden, Qujing, Yunnan province, China. Based on morpho-molecular analyses and previous literature, two new species of *Phragmocamarosporium* Wijayaw et al. (in the family *Lentitheciaceae*) and one new species of *Lonicericola* Phookamsak et al. (in the family *Parabambusicolaceae*) have been introduced. Besides, five new host/geographical records (*Botryosphaeria dothidea, Diplodia mutila*, and *D. seriata* in *Botryosphaeriaceae, Botryosphaeriales*, and *Angustimassarina populi* and *Shearia formosa* in *Amorosiaceae* and *Longiostiolaceae* respectively in the order *Pleosporales*) are herein reported. All the taxa are provided with illustrations and morphological descriptions. Furthermore, possibilities of revealing novel taxa of the respective genera and their distribution are also discussed.

## Materials and methods

### Sample collection, isolation, and identification

Samples were collected from aerial and ground litter (i.e., leaves, branches, and stems) of *Magnolia grandiflora* from September 2019 to June 2021 from Qujing Normal University garden in Yunnan, China. The specimens were stored in paper bags and transferred to the laboratory. The samples were examined with a stereomicroscope, and microscopic images of the samples were taken using a Canon EOS700D digital camera (Canon Inc., Ota, Tokyo, Japan) with a Nikon ECLIPSE Ni (Nikon Instruments Inc., Melville, NY, United States) compound microscope. Microcharacters were observed using a digital camera fitted onto a Nikon ECLIPSE 80i compound microscope. Measurements were per-formed with Tarosoft (R) Image Frame Work (v.0.9.7). More than 20 asci and ascospores (in sexual fungi) and more than 30 conidia and conidiogenous cells (in asexual fungi) were measured. Plates were prepared using the Adobe Photoshop CS6 (Adobe Systems, San Jose, CA, United States) software.

A single-spore isolation was carried out to isolate the taxa as described in Chomnunti et al. (2014), and we used water agar as the medium. A spore suspension was prepared using conidiomata or ascomata, and then the suspension was transferred with a sterile pipette onto the surface of a Petri dish with water agar. Germinated spores (approximately 12 h later) were transferred to a new potato dextrose agar (PDA) medium for purification. Dried specimens and living cultures were deposited at the herbarium and culture collection of Guizhou Medical University, Guizhou Province, China.

### DNA extraction, polymerase chain reaction amplification, and sequence analysis

The total genomic DNA of microfungi was extracted from fresh mycelia grown on PDA at 25–27°C using the Biospin Fungus Genomic DNA Extraction Kit (BioFlux^®^, Hangzhou, People's Republic of China) according to the manufacturer's instructions (Dai et al., 2019).

The primers used for amplification are listed in Table 1. PCR amplification conditions are those as followed by Dai et al. (2017). PCR products were sent for sequencing at Shanghai Sangon Biological Engineering Technology & Services Co. (Shanghai, People's Republic of China). All newly generated sequences are deposited in GenBank, and accession numbers are obtained (Table 2).

**Table 1:** Genes/loci and PCR primers used in this study.

**Gene region**	**Primers**	**References**
ITS	ITS5/ITS4	White et al. (1990)
LSU	LR0R/LR5	Vilgalys and Sun (1994)
SSU	NS1/NS4	White et al. (1990)
*tef1*	EF1-728F/EF-2	Rehner (2001)
	983F/2218R	Rehner (2001)
*rpb2*	fRPB2-5F/ fRPB2-7cR	Liu et al. (1999)

**Table 2:** Taxa used in the phylogenetic analyses and their corresponding GenBank numbers.

**Species**	**Strain no**.	**GenBank accession no**.				
		**SSU**	**LSU**	**ITS**	***tef1***	***rpb2***
*Aigialus grandis*	BCC 20000	GU479739	GU479775	–	GU479839	GU479814
*Aigialus mangrovis*	BCC 33563	GU479741	GU479776	–	GU479840	GU479815
*Aigialus parvus*	NFCCI-395	MK026763	MK026761	MK028710	MN520611	MN520612
*Aigialus rhizophorae*	BCC 33572	GU479745	GU479780	–	GU479844	GU479819
*Alfoldiavorosii* ^T^	REF116	MK589346	MK589354	JN859336	MK599320	–
*Amorocoelophoma camelliae*	NTUCC 18-097-1	MT071230	MT071279	MT112303	MT459143	MT743271
*Amorocoelophomacamelliae* ^T^	NTUCC 18-097-2	MT071231	MT071280	MT112304	MT459141	MT743272
*Amorocoelophomacassiae* ^T^	MFLUCC 17-2283	NG_065775	NG_066307	NR_163330	MK360041	MK434894
*Amorocoelophomaneoregeliae* ^T^	CBS 146820	–	NG_076726	MZ064410	MZ078247	MZ078193
*Amorosia littoralis*	NN 6654	AM292056	AM292055	AM292047	–	–
*Angustimassarinaacerina* ^T^	MFLUCC 14-0505	KP899123	KP888637	KP899132	KR075168	–
*Angustimassarinaalni* ^T^	MFLUCC 15-0184	KY548098	KY548097	KY548099	–	–
*Angustimassarinaarezzoensis* ^T^	MFLUCC 13-0578	KY501113	KY496722	KY496743	KY514392	–
*Angustimassarinacamporesii* ^T^	MFLU 18-0057	MN244173	MN244167	NR_168223	–	–
*Angustimassarinacoryli* ^T^	MFLUCC 14-0981	–	MF167432	MF167431	MF167433	–
*Angustimassarinaitalica* ^T^	MFLUCC15-0082	KY501124	KY496736	KY496756	KY514400	–
*Angustimassarinalonicerae* ^T^	MFLUCC15-0087	–	KY496724	KY496759	–	–
*Angustimassarina populi*	MFLUCC 17-1217	MG812610	MG812609	MG763958	MG812528	–
*Angustimassarina populi*	MFLUCC 17-1069	MF409165	MF409166	MF409170	MF409163	–
*Angustimassarina populi*	MFLUCC 21-0178	OL824798	OL813502	OM212462	–	–
*Angustimassarina populi*	MFLUCC 21-0175	OL824797	OL813501	OM212461	–	–
*Angustimassarinapopuli* ^T^	MFLUCC 13-0034	KP899128	KP888642	KP899137	KR075164	–
*Angustimassarina populi*	GMBCC1177	OM855611	OM855597	OM855588	OM857552	–
*Angustimassarina premilcurensis*	MFLUCC 15-0074	–	KY496725	KY496745	–	KY514404
*Angustimassarina quercicola*	MFLUCC 14-0506	KP899124	KP888638	KP899133	KR075169	–
*Angustimassarina rosarum*	MFLUCC 15-0080	–	MG828985	MG828869	–	–
*Angustimassarina rosarum*	MFLUCC 17-2155	MT226662	MT214543	MT310590	MT394726	MT394678
*Angustimassarina sylvatica*	MFLUCC 18-0550	MK314097	MK307844	MK307843	MK360181	–
*Aquastromamagniostiolata* ^T^	CBS 139680	AB797220	AB807510	LC014540	AB808486	–
*Aquasubmersa japonica*	KT2813	LC061581	LC061586	LC061591	LC194383	LC194420
*Aquasubmersajaponica* ^T^	KT2862	LC061582	LC061587	LC061592	LC194384	LC194421
*Aquasubmersa japonica*	KT2863	LC061583	LC061588	LC061593	LC194385	LC194422
*Aquilomycespatris* ^T^	CBS 135661	KP184077	KP184041	KP184002	–	–
*Aquilomycesrebunensis* ^T^	CBS 139684	AB797252	AB807542	AB809630	AB808518	–
*Bambusicolamassarinia* ^T^	MFLUCC 11-0389	JX442041	JX442037	NR_121548	KP761725	KP761716
*Botryosphaeriadothidea* ^T^	CBS 115476	NG_062738	NG_027577	NR_111146	–	–
*Botryosphaeria dothidea*	AFTOL-ID 946	–	DQ678051	–	DQ767637	DQ677944
*Botryosphaeria dothidea*	MFLUCC 16-0936	MT177977	MT177950	MT177923	–	MT432204
*Botryosphaeria dothidea*	GMBCC1179	OM855612	OM855598	OM855589	OM857553	–
*Clypeoloculusakitaensis* ^T^	CBS 139681	AB797253	AB807543	AB809631	AB808519	–
*Crassiperidiumoctosporum* ^T^	KT 2144	LC373084	LC373108	LC373096	LC373120	LC373132
*Crassiperidium octosporum*	KT 2894	LC373085	LC373109	LC373097	LC373121	LC373133
*Crassiperidium octosporum*	KT 3008	LC373086	LC373110	LC373098	LC373122	LC373134
*Crassiperidium quadrisporum*	KT 2798-1	LC373094	LC373118	LC373106	LC373130	LC373142
*Crassiperidiumquadrisporum* ^T^	KT 2798-2	LC373095	LC373119	LC373107	LC373131	LC373143
*Cucitellaopali* ^T^	CBS 142405	MF795837	MF795754	MF795754	MF795843	MF795796
*Darksideaalpha* ^T^	CBS 135650	NG_061189	NG_059126	NR_137619	KP184166	–
*Darksideabeta* ^T^	CBS 135637	KP184074	KP184023	NR_137957	KP184189	–
*Darksideadelta* ^T^	CBS 135638	KP184069	KP184024	NR_137075	KP184184	–
*Diatrype disciformis*	AFTOL-ID 927	DQ471012	DQ470964	–	DQ471085	DQ470915
*Diplodiamutila* ^T^	CBS 136014	–	–	KJ361837	–	–
*Diplodia mutila*	CBS 230.30	–	EU673265	MW810264	–	–
*Diplodia mutila*	FR36	–	KY554740	KY554742	–	–
*Diplodia mutila*	AFTOL-ID 1572	DQ678012	DQ377863	KU198424	DQ677907	DQ677960
*Diplodia mutila*	GMBCC1173	OM855610	OM855596	OM855587	OM857551	–
*Diplodia seriata*	N/A	OM855608	OM855594	OM855585	–	–
*Diplodiaseriata* ^T^	CBS 112555	NG_062751	KF766327	AY259094	–	–
*Diplodia seriata*	CBS 119049	EU673216	EU673266	DQ458889	–	–
*Diplodia seriata*	MZ-F47	MG785011	MG720320	KU942441	–	–
*Diplodia seriata*	Mz-F45	MG785010	MG720319	KU942440	–	–
*Diplodia seriata*	GMBCC1175	OM855609	OM855595	OM855586	–	–
*Dothidotthiarobiniae* ^T^	MFLUCC 16-1175	MK751762	MK751817	MK751727	MK908017	MK920237
*Falciformispora lignatilis*	BCC 21117	GU371834	GU371826	KF432942	GU371819	–
*Falciformisporasenegalensis* ^T^	CBS 196.79	KF015636	KF015631	KF015673	KF015687	KF015717
*Falciformisporatompkinsii* ^T^	CBS 200.79	KF015639	KF015625	NR_132041	KF015685	KF015719
*Fenestellamedia* ^T^	CBS 144860	MK356326	MK356285	MK356285	MK357558	MK357515
*Graphostroma platystoma*	CBS 270.87	DQ836900	DQ836906	JX658535	DQ836915	DQ836893
*Halobyssotheciumobiones* ^T^	MFLUCC 15-0381	MH376745	MH376744	MH377060	MH376746	–
*Katumotoabambusicola* ^T^	KT 1517a	AB524454	AB524595	LC014560	AB539108	AB539095
*Keissleriellabreviasca* ^T^	KT 649	AB797298	AB807588	AB811455	AB808567	–
*Keissleriellacirsii* ^T^	MFLUCC 16-0454	KY497782	NG_059776	NR_155248	KY497786	–
*Keissleriellaquadriseptata* ^T^	KT 2292	AB797303	AB807593	AB811456	AB808572	–
*Keissleriella quadriseptata*	MFLU 19-2871	MT214957	MT183478	MT185515	MT454026	MT432229
*Lentitheciumaquaticum* ^T^	CBS 123099	GU296156	GU301823	MH863276	GU349068	FJ795455
*Lentitheciumclioninum* ^T^	KT 1149A	AB797250	AB807540	LC014566	AB808515	–
*Lentithecium clioninum*	KT 1220	AB797251	AB807541	LC014567	AB808516	–
*Lentithecium pseudoclioninum*	KT 1111	AB797254	AB807544	AB809632	AB808520	–
*Lentitheciumpseudoclioninum* ^T^	KT 1113	AB797255	AB807545	AB809633	AB808521	–
*Longiostiolumtectonae* ^T^	MFLUCC 12-0562	NG_061231	KU764700	NR_148100	–	–
*Lonicericolafuyuanensis* ^T^	MFLU 19-2850	MN917867	MN917865	MN917866	MN938324	–
*Lonicericolahyaloseptispora* ^T^	KUMCC 18-0149	MK098203	NG_066434	NR_164294	–	–
*Lonicericola hyaloseptispora*	KUMCC 18-0150	MK098206	MK098200	MK098194	MK098210	–
*Lonicericolaqujingensis* ^T^	GMBCC1178	OM855616	OM855602	OM855593	OM857556	–
*Multiloculariabambusae* ^T^	MFLUCC 11-0180	KU693442	KU693438	KU693446	–	
*Multiseptosporathailandica* ^T^	MFLUCC 11-0183	KP753955	KP744490	KP744447	–	–
*Multiseptospora thailandica*	MFLUCC 11-0204	KU693444	KU693440	KU693447	KU705659	KU705661
*Multiseptospora thailandica*	MFLUCC 12-0006	KU693445	KU693441	KU693448	KU705660	KU705662
*Murilentithecium clematidis*	MFLUCC 14-0561	KM408760	KM408758	KM408756	KM454444	KM454446
*Murilentitheciumclematidis* ^T^	MFLUCC 14-0562	NG_061185	KM408759	NR_154174	KM454445	KM454447
*Murilentitheciumlonicerae* ^T^	MFLUCC 18-0675	MK214376	MK214373	MK214370	MK214379	–
*Murilentitheciumrosae* ^T^	MFLUCC 15-0044	MG829137	MG829030	MG828920	–	–
*Neoaquastromabauhiniae* ^T^	MFLUCC 16-0398	MH023315	MH023319	MH025952	MH028247	MH028251
*Neoaquastroma bauhiniae*	MFLUCC 17-2205	MH023316	MH023320	MH025953	MH028248	MH028252
*Neoaquastromacylindricum* ^T^	MFLUCC 19-0489	MN473048	MN473054	MN473060	MN481600	–
*Neoaquastromakrabiense* ^T^	MFLUCC 16-0419	MH023317	MH023321	MH025954	MH028249	MH028253
*Neoophiosphaerellasasicola* ^T^	KT 1706	AB524458	AB524599	LC014577	AB539111	–
*Occultibambusachiangraiensis* ^T^	MFLUCC 16-0380	NG_062421	KX655546	–	KX655561	KX655566
*Occultibambusajonesii* ^T^	GZCC 16-0117	NG_065104	NG_066381	–	KY814756	KY814758
*Palmiascomagregariascomum* ^T^	MFLUCC 11-0175	KP753958	KP744495	KP744452	–	KP998466
*Parabambusicola bambusina*	KH 4321	AB797247	AB807537	LC014579	AB808512	–
*Parabambusicola bambusina*	KH 139	AB797246	AB807536	LC014578	AB808511	–
*Parabambusicola bambusina*	KH 2637	AB797248	AB807538	LC014580	AB808513	–
*Parabambusicolathysanolaenae* ^T^	KUMCC 18-0147	MK098205	NG_066435	NR_164044	MK098209	–
*Parabambusicola thysanolaenae*	KUMCC 18-0148	MK098202	MK098198	MK098193	MK098211	–
*Parafenestella rosacearum*	FM1	MK356327	MK356313	MK356313	MK357585	MK357541
*Parathyridariaramulicola* ^T^	CBS 141479	KX650514	KX650565	KX650565	KX650536	KX650584
*Paratrimmatostromakunmingensis* ^T^	HKAS 102224A	MK098204	MK098196	MK098192	MK098208	–
*Paratrimmatostroma kunmingensis*	HKAS 102224B	MK098207	MK098201	MK098195	–	–
*Phaeosphaeriachiangraina* ^T^	MFLUCC 13-0231	KM434289	KM434280	KM434270	KM434298	KM434307
*Phaeosphaeriamusae* ^T^	MFLUCC 11-0133	KM434287	KM434277	KM434267	KM434296	KM434304
*Phaeosphaeriathysanolaenicola* ^T^	MFLUCC 10-0563	KM434286	KM434276	KM434266	KM434295	KM43430
*Phragmocamarosporium hederae*	KUMCC 18-0165	MK214375	MK214372	MK214369	MK214378	–
*Phragmocamarosporiumhederae* ^T^	MFLUCC 13-0552	KP842918	KP842915	–	–	–
*Phragmocamarosporiummagnoliae* ^T^	GMBCC1180	OM855614	OM855600	OM855591	OM857555	–
*Phragmocamarosporium magnoliae*	GMBCC1041	ON364114	ON364110	ON364112	ON375375	–
*Phragmocamarosporiumplatani* ^T^	MFLUCC 14-1191	KP842919	KP842916	KP852526	–	–
*Phragmocamarosporiumqujingensis* ^T^	GMBCC1176	OM855613	OM855599	OM855590	OM857554	–
*Phragmocamarosporium qujingensis*	GMBCC1044	ON364113	ON364109	ON364111	ON375374	–
*Phragmocamarosporiumrosae* ^T^	MFLUCC 17-0797	MG829156	NG_059874	–	MG829225	–
*Poaceascomaaquaticum* ^T^	MFLUCC 14-0048	KT324691	KT324690	–	–	KT373846
*Poaceascomahelicoides* ^T^	MFLUCC 11-0136	KP998463	KP998462	KP998459	KP998461	KP998460
*Pseudomonodictystectonae* ^T^	MFLUCC 12-0552	KT285574	KT285573	–	KT285571	KT285572
*Roussoella hysterioides*	CBS 546.94	AY642528	KF443381	KF443405	KF443399	KF443392
*Roussoella pustulans*	MAFF 239637	AB524482	AB524623	KJ474830	AB539116	AB539103
*Salsuginea ramicola*	KT 2597.1	GU479767	GU479800	–	GU479861	GU479833
*Salsuginea ramicola*	KT 2597.2	GU479768	GU479801	–	GU479862	GU479834
*Sclerostagonospora cycadis*	CBS 291.76	–	–	KR611890	–	–
*Setoseptoria arundinacea*	KT 552	AB797284	AB807574	LC014594	AB808550	–
*Setoseptoria arundinacea*	KT 600	AB797285	AB807575	LC014595	AB808551	–
*Setoseptoriamagniarundinacea* ^T^	KT 1174	AB797286	AB807576	LC014596	AB808552	–
*Shearia formosa*	MFLUCC 20-0017	MT159631	MT159619	MT159625	MT159602	MT159608
*Shearia formosa*	MFLUCC 20-0018	MT159633	MT159621	MT159627	MT159604	MT159610
*Sheariaformosa* ^T^	MFLUCC 20-0019	MT159632	MT159620	MT159626	MT159603	MT159609
*Shearia formosa*	GMBCC1172	OM855615	OM855601	OM855592	–	–
*Sordaria fimicola*	AFTOL-ID 216	AH007748	FR774289	DQ518178	DQ518175	DQ368647
*Thyridariabroussonetiae* ^T^	TB1	KX650515	KX650569	KX650569	KX650539	KX650586
*Thyrostromalycii* ^T^	MFLUCC 16-1170	MK751769	MK751824	MK751734	MK908024	MK920241
*Thyrostromatiliae* ^T^	MFLUCC 16-1178	MK751773	MK751828	MK751738	MK908028	MK920245
*Tingoldiagograminicola* ^T^	KH 68	AB521726	AB521743	LC014598	AB808561	–
*Tingoldiago graminicola*	KH 155	AB521728	AB521745	LC014599	AB808562	–
*Tingoldiago graminicola*	KT 891	AB521727	AB521744	LC014600	AB808563	–
*Towysporaaestuari* ^T^	MFLUCC 15-1274	NG_061225	NG_060798	NR_148095	–	–
*Trematosphaeriapertusa* ^T^	CBS 122368	FJ201991	FJ201990	NR_132040	KF015701	FJ795476
*Tzeananiataiwanensis* ^T^	NTUCC 17-005	MH461126	MH461120	MH461123	MH461130	MH461128
*Tzeanania taiwanensis*	NTUCC 17-006	MH461127	MH461121	MH461124	MH461131	MH461129

The newly generated sequences are indicated in bold and ex-type strains are with “T”.

AFTOL, Assembling the Fungal Tree of Life culture collection; BBH, BIOTEC Bangkok Herbarium, Thailand; BCC, Belgian Coordinated Collections of Microorganisms; BCC, BIOTEC Culture Collection, Thailand; CBS, Culture collection of the Centraal bureau voor Schimmel cultures, Fungal Biodiversity Center, Utrecht, The Netherlands; FM, Felix Slavik Straße (Marchfeldkanalweg); FR, France; GMBCC, Guizhou Medical University Culture Collection; GZCC, Guizhou Culture Collection; HKAS, Fungarium of the Cryptogams Kunming Institute of Botany, Academia Sinica; KH, K. Hirayama; KT, K. Tanaka; KUMCC, Kunming Culture Collection; MAFF, Ministry of Agriculture, Forestry and Fisheries, Japan; MFLU, Herbarium of Mae Fah Luang University; MFLUCC, Mae Fah Luang University Culture Collection; Mz-F, Chilean isolates sequenced in Díaz et al. (2018); NN, NovoNordisk culture collection (now Novozymes, Bagsvaerd, Denmark); NTUCC, National Taiwan University Culture Collection; REF, Root Endophytic Fungi; TB, Thyridaria broussonetiae.

### Sequencing and sequence alignment

Sequences generated from different primers of non-translated loci and protein-coding regions are analyzed with other sequences retrieved from GenBank (Table 2). Sequences with high similarity indices were determined by a BLAST search to find closest matches with taxa in *Dothideomycetes* and from recently published data (e.g., Thambugala et al., 2015; Tibpromma et al., 2017; Wanasinghe et al., 2018, 2020; Hyde et al., 2020). The multiple alignments of all consensus sequences, as well as the reference sequences, were automatically generated with MAFFT v. 7 (Katoh et al., 2017), and were improved manually when necessary using BioEdit v. 7.0.5.2 (Hall, 1999).

### Phylogenetic analyses

#### Analysis 1 (SSU, LSU, ITS, *tef1*, and *rpb2* multi-sequence analyses of *Amorosiaceae, Botryosphaeriaceae, Lentitheciaceae, Longiostiolaceae*, and *Parabambusicolaceae*)

Single-locus data sets were examined for topological incongruence among loci for members of the relevant families. Conflict-free alignments were combined in to the final multi-gene dataset for analyses using BioEdit and concatenated into a multi-locus alignment that was subjected to maximum-likelihood (ML) and Bayesian (BI) phylogenetic analyses. The CIPRES Science Gateway platform (Miller et al., 2010) was used to perform RAxML and Bayesian analyses. ML analyses were performed with RAxML-HPC2 on XSEDE v. 8.2.10 (Stamatakis, 2014) using a GTR + I + G model with 1,000 bootstrap repetitions. Evolutionary models for Bayesian analysis were selected independently for each locus using MrModeltest v. 2.3 (Nylander et al., 2008) under the Akaike Information Criterion (AIC) implemented in both PAUP v. 4.0b10, and GTR + I + G was selected as the best-fit model for all three analyses. MrBayes analyses were performed setting GTR + I + G, 2 M generations, sampling every 100th generation and ending the run automatically when the standard deviation of split frequencies dropped below 0.01 with a burn-in fraction of 0.25.

#### Analysis 2 (ITS and *tef1* sequence analyses of *Botryosphaeria sensu stricto*)

The ITS and *tef1* data sets were examined for topological incongruence among loci for selected members of *Botryosphaeria*. Conflict-free alignments were concatenated into a multi-locus alignment that was subjected to maximum-likelihood (ML) phylogenetic analysis. The CIPRES Science Gateway platform (Miller et al., 2010) was used to perform RAxML. ML analyses were performed with RAxML-HPC2 on XSEDE v. 8.2.10 (Stamatakis, 2014) using the GTR + I + G model with 1,000 bootstrap repetitions. A Bayesian analysis was performed using SYM + I + G for ITS and GTR + I for *tef1* in the final command with 1 M generations. Sampling was conducted on every 100th generation, ending the run automatically when the standard deviation of split frequencies dropped below 0.01 with a burn-in fraction of 0.25.

#### Analysis 3 (ITS and *tef1* sequence analyses of *Diplodia sensu stricto*)

The ITS and *tef1* data sets were examined for topological incongruence among loci for selected members of *Diplodia*. Conflict-free alignments were concatenated into a multilocus alignment that was subjected to maximum-likelihood (ML) phylogenetic analysis. The CIPRES Science Gateway platform (Miller et al., 2010) was used to perform RAxML. ML analyses were performed with RAxML-HPC2 on XSEDE v. 8.2.10 (Stamatakis, 2014) using the GTR + I + G model with 1,000 bootstrap repetitions. A Bayesian analysis was performed using GTR + I + G for ITS and HKY + G for *tef1* in the final command with 1 M generations. Sampling was conducted on every 100th generation, ending the run automatically when the standard deviation of split frequencies dropped below 0.01 with a burn-in fraction of 0.25.

#### Analysis 4 (ITS, LSU, SSU, and *tef1* sequence analyses of *Amorosiaceae*)

The ITS, LSU, SSU, and *tef1* data sets were examined for topological incongruence among loci for selected members of *Amorosiaceae*. Conflict-free alignments were concatenated into a multi-locus alignment that was subjected to maximum-likelihood (ML) phylogenetic analysis. The CIPRES Science Gateway platform (Miller et al., 2010) was used to perform RAxML. ML analyses were performed with RAxML-HPC2 on XSEDE v. 8.2.10 (Stamatakis, 2014) using the GTR + I + G model with 1,000 bootstrap repetitions. MrBayes analyses were performed setting GTR + I + G, 1 M generations, sampling every 100th generation, ending the run automatically when the standard deviation of split frequencies dropped below 0.01 with a burn-in fraction of 0.25.

#### Analysis 5 (SSU, LSU, *tef1*, and ITS sequence analyses of *Lentitheciaceae*)

Raw sequences were combined using SeqMan and subjected to BLAST in GenBank. The SSU, LSU, *tef1*, and ITS sequence data closely related to our taxa were retrieved from the NCBI GenBank and are listed in Table 1. Single gene sequence alignment was generated with the MAFFT v. 7 online program (http://mafft.cbrc.jp/alignment/server/) (Katoh et al., 2017). FASTA alignment formats were changed to PHYLIP and NEXUS formats with Aliview 2.11. The single-gene datasets were examined for topological incongruence among loci and the conflict-free alignments were concatenated into a multi-locus alignment that was subjected to ML and BI phylogenetic analyses. The CIPRES Science Gateway platform (Miller et al., 2010) was used to perform RAxML. ML analyses were conducted with RAxML-HPC2 on XSEDE v. 8.2.10 (Stamatakis, 2014) using GTR + I + G model with 1,000 bootstrap repetitions. MrBayes analyses were performed setting GTR + I + G, two parallel runs were conducted, using the default settings, six simultaneous Markov chains were run for 1 M generations, and trees were sampled every 100th generation. The run ended automatically when the standard deviation of split frequencies dropped below 0.01 with a burn-in fraction of 0.2. Phylograms were visualized with the FigTree v1.4.0 program (Rambaut, 2012) and reorganized in Microsoft PowerPoint (2019) and Adobe Illustrator^®^ CS5 (Version 15.0.0, Adobe^®^, San Jose, CA).

## Results

### Phylogenetic analyses

**Figure 1:**
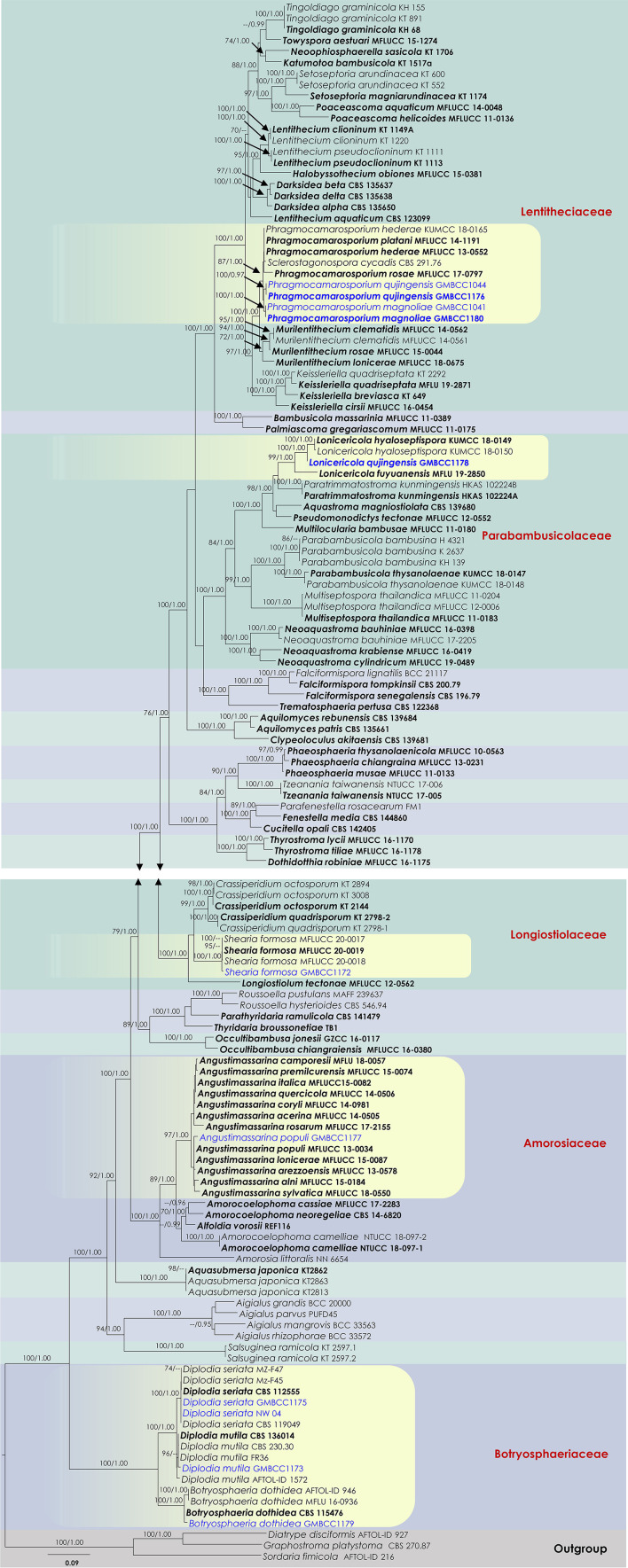
RAxML tree based on a combined dataset of partial SSU, LSU, ITS, *tef1*, and *rpb2* DNA sequence analyses. Bootstrap support values for ML equal to or >70%, Bayesian posterior probabilities (BYPP) equal to or >0.95, are shown as ML/BI above the nodes. The type of strains is in bold, and new isolates are in blue. The scale bar represents the expected number of nucleotide substitutions per site.

Analysis 1 (SSU, LSU, ITS, *tef1*, and *rpb2* multi-sequence analyses of *Amorosiaceae, Botryosphaeriaceae, Lentitheciaceae, Longiostiolaceae*, and *Parabambusicolaceae*): The concatenated dataset (SSU, LSU, ITS, *tef1*, and *rpb2* loci) contained 140 isolates, and the tree was rooted to *Diatrype disciformis* (AFTOL-ID 927), *Graphostroma platystoma* (CBS 270.87), and *Sordaria fimicola* (AFTOL-ID 216). The final alignment contained 4,399 characters used for the phylogenetic analyses, including alignment gaps, which were treated as missing data. The RAxML analysis of the combined datasets yielded a best-scoring tree with a final ML optimization likelihood value of −62,337.358653. The matrix had 2,549 distinct alignment patterns, with 26.87% undetermined characters or gaps. Parameters for the GTR + I + G model of the combined amplicons were as follows: estimated base frequencies; A = 0.241265, C = 0.251518, G = 0.269739, and T = 0.237478; substitution rates AC = 1.482469, AG = 3.530918, AT = 1.543663, CG = 1.180062, CT = 7.309441, and GT = 1; proportion of invariable sites I = 0.428895; gamma distribution shape parameter α = 0.610427. Based on the results of MrModel Test, dirichlet base frequencies and the GTR + I + G model were used for the Bayesian analysis. The Bayesian analyses generated 9,001 trees (saved every 100th generation), from which 6,751 were sampled after 25% of the trees were discarded as burn-ins. The alignment contained a total of 2,551 unique site patterns. In the combined multigene phylogenetic analysis, *Phragmocamarosporium* species (*P. hederae, P. platani*, and *P. rosae*) clustered in one clade (87% ML/1 PP, Figure 1), sister to *Murilentithecium* (100 ML/1 PP, Figure 1). The strain “*Sclerostagonospora cycadis*” (CBS 291.76) was also nested with *Phragmocamarosporium* species. The two strains of *Phragmocamarosporium hederae* (MFLUCC 13-0552 and KUMCC 18-0165) were not monophyletic. The four strains of *Phragmocamarosporium* (GMBCC1041, GMBCC1044, GMBCC1176, and GMBCC1180) isolated in this study formed a basal terminal clade in *Phragmocamarosporium* with <70% ML and <0.95 BYPP both single locus and concatenated datasets. In this clade, GMBCC1044 and GMBCC1176 constituted a monophyletic clade with 100 ML/1 PP support values (Figure 1). GMBCC1041 and GMBCC1180 also displayed a strongly supported monophyletic lineage (100% ML/1 PP, Figure 1). These two new lineages are presented here as new species, *viz*., *Phragmocamarosporium magnoliae* sp. nov. (GMBCC1041 and GMBCC1180) and *P. qujingensis* sp. nov. (GMBCC1044 and GMBCC1176).

The family *Parabambusicolaceae* was resolved into eight distinct clades representing species in *Aquastroma, Lonicericola, Multilocularia, Multiseptospora, Neoaquastroma, Parabambusicola, Paratrimmatostroma*, and *Pseudomonodictys*. Our new strain, GMBCC1178, constituted a strong monophyletic relationship with *Lonicericola fuyuanensis* (MFLU 19-2850) and *L. hyaloseptispora* (KUMCC 18-0150 and KUMCC 18-0149). This new lineage (GMBCC1178) is presented here as the new species *Lonicericola qujingensis* sp. nov.

The *Shearia formosa* (GMBCC1172) isolated in this study nested in a well-supported clade (100% ML/1 BYPP) with other isolates of *S. formosa* (MFLUCC 20-0017, MFLUCC 20-0018, and MFLUCC 20-0019), which were used by Wanasinghe et al. (2020) to describe the species, therefore confirming the identification of the studied species. The family *Amorosiaceae* is composed of four clades, which correspond to known genera *Alfoldia, Amorocoelophoma, Amorosia*, and *Angustimassarina*. Our new strain, GMBCC1177, grouped with another 12 *Angustimassarina* strains with 97% ML and 1 BYPP statistical support values. However, the interspecific relationships of these *Angustimassarina* species have not received a clear phylogenetic resolution. Our new isolate has a close phylogenetic affinity to *Angustimassarina populi*.

Analysis 2 (ITS and *tef1* sequence analyses of *Botryosphaeria sensu stricto*): The concatenated dataset (ITS and *tef1* loci) contained 33 isolates, and the tree was rooted to *Macrophomina phaseolina* (CBS 227.33). The final alignment contained 761 characters used for phylogenetic analyses, including alignment gaps. The RAxML analysis of the combined dataset yielded a best-scoring tree with a final ML optimization likelihood value of −1,861.242225. The matrix had 161 distinct alignment patterns, with 6.84 % undetermined characters or gaps. Parameters for the GTR + I + G model of the combined amplicons were as follows: estimated base frequencies; A = 0.210798, C = 0.292947, G = 0.259679, and T = 0.236576; substitution rates AC = 0.435178, AG = 1.502643, AT = 1.277448, CG = 0.440383, CT = 4.352625, and GT = 1; proportion of invariable site I = 0.701032; gamma distribution shape parameter α = 0.87371. In the combined sequence data analyses of the ITS and *tef1* loci, our new strain, GMBCC1179, clustered with 16 other strains of *Botryosphaeria dothidea* (Supplementary Figure 1). Two strains of *Botryosphaeria auasmontanum* (MFLUCC 15-0923 and MFLUCC 17-1071) also grouped in *B. dothidea*. However, the *Botryosphaeria dothidea* clade is statistically not well-supported.

Analysis 3 (ITS and *tef1* sequence analyses of *Diplodia sensu stricto*): The concatenated ITS and *tef1* loci contained 67 isolates, and the tree was rooted to *Lasiodiplodia lignicola* (MFLUCC 11-0656). The final alignment contained 882 characters used for the phylogenetic analyses, including alignment gaps. The RAxML analysis of the combined dataset yielded a best-scoring tree with a final ML optimization likelihood value of −3,628.405258. The matrix had 321 distinct alignment patterns, with 12.65 % undetermined characters or gaps. Parameters for the GTR + I + G model of the combined amplicons were as follows: estimated base frequencies; A = 0.206552, C = 0.298475, G = 0.261435, and T = 0.233538; substitution rates AC = 1.148622, AG = 3.343902, AT = 1.039052, CG = 1.718372, CT = 4.817617, and GT = 1; proportion of invariable site I = 0.441349; gamma distribution shape parameter α = 0.65846. In our analysis of selected *Diplodia* species, the new strain GMBCC1173 clustered with *Diplodia mutila* (MFLUCC 15-0918, CBS 230.30, CBS 112553, CBS 136014, and MFLUCC 15-0917). Particularly, GMBCC1173 has a close phylogenetic affinity to MFLUCC 15-0917, which was introduced by Dissanayake et al. (2017) from Italy on *Acer negundo*. The two collections (GMBCC1175 and NW04) isolated in this study formed a basal terminal lineage in the *Diplodia seriata* clade that includes thirteen strains. This *Diplodia seriata* clade also did not receive a strong phylogenetic support (Supplementary Figure 2).

**Figure 2:**
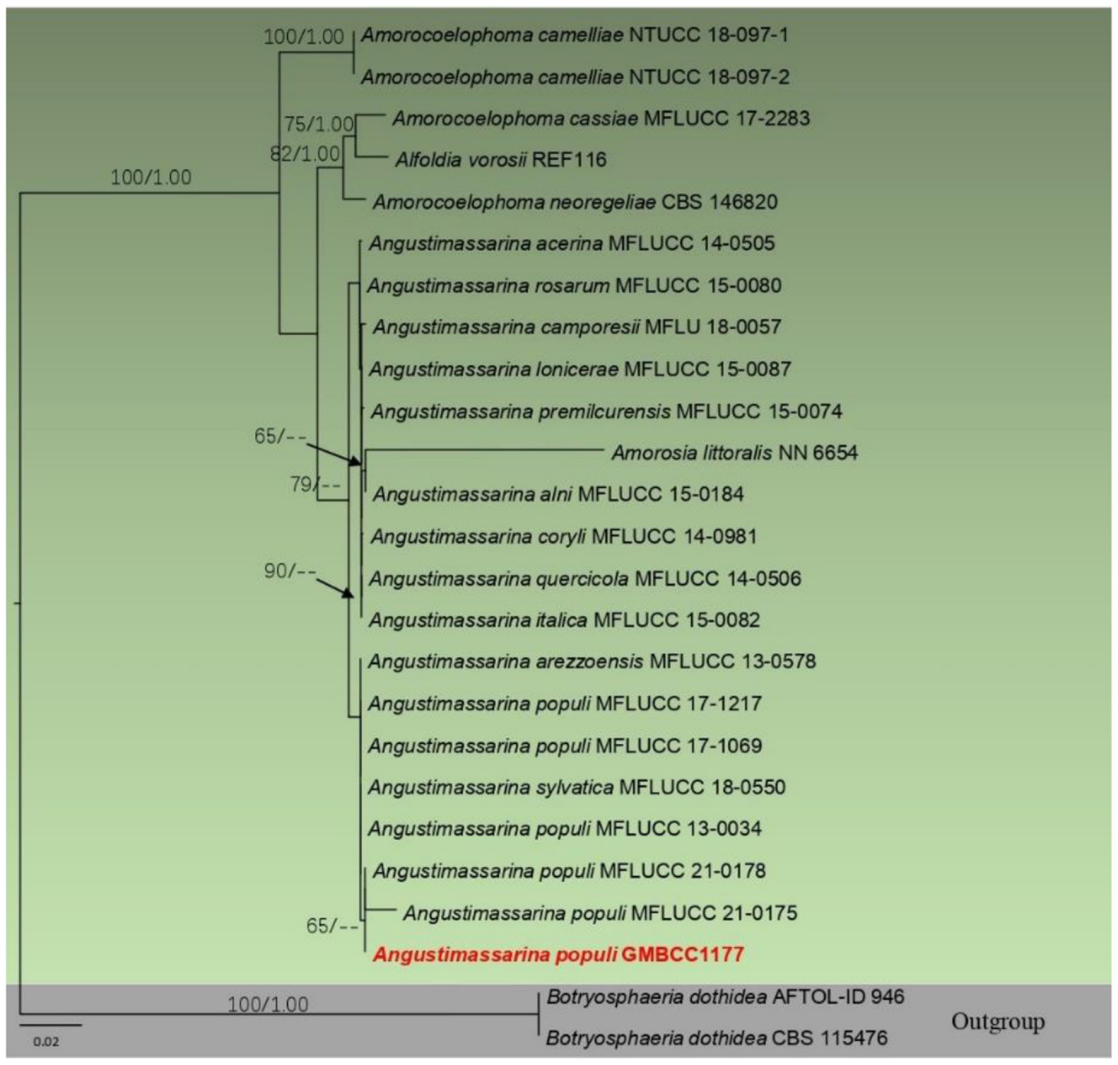
Phylogram generated from maximum likelihood analysis based on combined SSU, LSU, *tef1*, and ITS partial sequence data. Bootstrap support values for ML equal to or >50% and BYPP values equal to or >0.9 are given above the nodes. The newly generated sequence is in red.

Analysis 4 (ITS, LSU, SSU, and *tef1* sequence analyses of *Amorosiaceae*): Twenty-five strains are included in the sequence analysis and comprise 2,106 characters with gaps. A single gene analysis was carried out and compared with each species to compare the topology of the tree and clade stability. *Botryosphaeria dothidea* (CBS 115476 and AFTOL-ID 946) was used as the outgroup taxon. The tree topology of the ML analysis was similar to the BYPP. The best-scoring RAxML tree with a final likelihood value of −5144.567766 is presented. The matrix had 247 distinct alignment patterns, with 18.63% of undetermined characters or gaps. Estimated base frequencies were as follows: A = 0.243552, C = 0.248710, G = 0.270808, and T = 0.236930; substitution rates AC = 0.682136, AG = 1.407781, AT = 1.275132, CG = 0.809575, CT = 6.927148, and GT = 1; gamma distribution shape parameter alpha = 0.654596 (Figure 2). The family *Amorosiaceae* is composed of four clades, which correspond to known genera *Alfoldia, Amorocoelophoma, Amorosia*, and *Angustimassarina*. Our new strain, GMBCC1177, grouped with another *Angustimassarina* strains with low statistical support values (Figure 2). However, the interspecific relationships of these *Angustimassarina* species have not received a clear phylogenetic resolution. The strain GMBCC1177 showed a close phylogenetic affinity with *Angustimassarina* populi strains clustered together with *A. arezzoensis* (MFLUCC 13-0578) and *A. sylvatica* (MFLUCC 18-0550).

**Figure 3:**
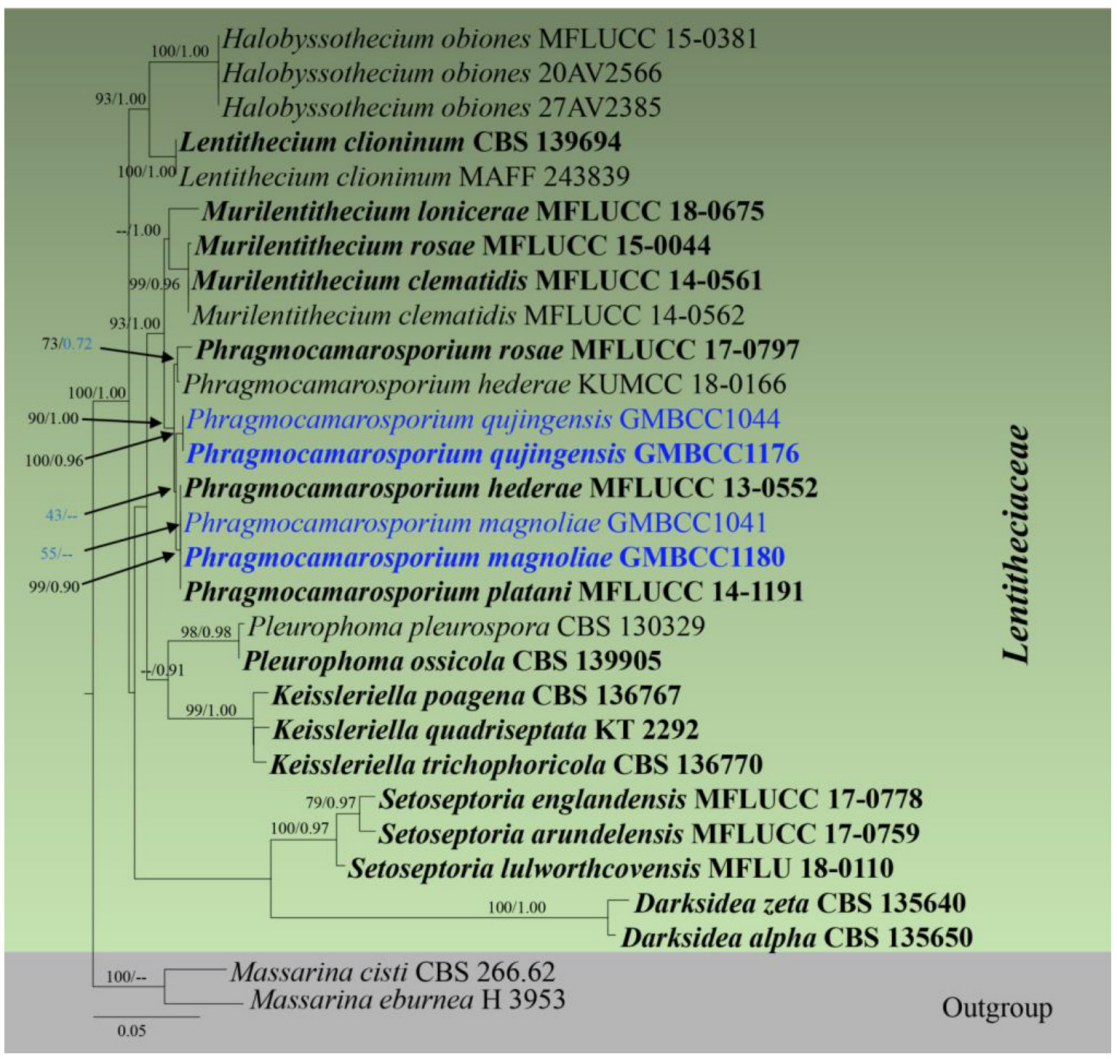
RAxML tree based on a combined dataset of partial SSU, LSU, *tef1*, and ITS DNA sequence analyses. Bootstrap support values for ML equal to or >50% and Bayesian posterior probabilities (BYPP) equal to or >0.9 are shown as ML/BI above/below the nodes (except for those values in blue in *Phragmocamarosporium* clade). The type of strains is in bold, and new isolates are in blue. The scale bar represents the expected number of nucleotide substitutions per site.

Analysis 5 (SSU, LSU, *tef1*, and ITS sequence analyses of *Lentitheciaceae*): Twenty-nine strains are included in the sequence analysis and comprise 3,086 characters with gaps. A single gene analysis was carried out and compared with each species to compare the topology of the tree and clade stability. *Massarina cisti* (CBS 266.62) and *Massarina eburnea* (H 3953) are used as outgroup taxa. The tree topology of the ML analysis was similar to the BYPP. The best-scoring RAxML tree with a final likelihood value of −10,433.446109 is presented. The matrix had 492 distinct alignment patterns, with 25.16% of undetermined characters or gaps. Estimated base frequencies were as follows: A = 0.237977, C = 0.252206, G = 0.270764, and T = 0.239052; substitution rates AC = 0.947399, AG = 1.742628, AT = 0.987565, CG = 1.240193, CT = 6.545005, and GT = 1; gamma distribution shape parameter alpha = 0.608667 (Figure 3). The family *Lentitheciaceae* comprises eight genera that show distinct phylogenetic lineages (Figure 3). Separation of *Phragmocamarosporium* species is agreement with morphological evidence (**Table 4**), but the new collections GMBCC1180 and GMBC1041 clustered with ex-types of *P. hederae* and *P. platani* with low bootstrap values (which are indicated in blue in Figure 3). The ex-types of *P. hederae* and *P. platani* are lacking ITS loci in the GenBank; thus, we suggest that including more gene regions of these two species will facilitate a better understanding of intraspecific segregation. However, here, we follow the morphological evidence to introduce novel species (i.e., *P. magnoliae*) (see below under the Section Taxonomy).

GMBCC1176 and GMBCC1044, are grouped as the basal clade to the clade that comprises ex-types of *P. hederae, P. magnoliae*, and *P. platani* (Figure 3). Both strains were generated from morphologically similar collections and thus introduced as a new species, i.e., *Phragmocamarosporium qujingensis*.

The strain named *Phragmocamarosporium hederae* (KUMCC 18-0165) clustered with the ex-type of *P. rosae* (MFLUCC 17-0797) but was distinct from the ex-type of *P. hederae*. This strain was named mistakenly as *Phragmocamarosporium hederae* and thus needs an extensive study to confirm if it warrants a novel species.

### Taxonomy

In this section, we introduce three new pleosporalean species from *M. grandiflora* in Qujing Normal University, Qujing, Yunnan Province, China. Moreover, five (three in *Botryosphaeriales* and two in *Pleosporales*) species are reported as a new host and geographical records from *M. grandiflora* and China, respectively.

***Botryosphaeriales*** C.L. Schoch, Crous, & Shoemaker (2007)

***Botryosphaeriaceae*** Theiss. & Syd. [as “Botryosphaeriacae”], Annls mycol. 16(1/2): 16 (1918)

#### Notes

*Botryosphaeriaceae* (in *Botryosphaeriales*) is an important family that comprises a broad range of life modes such as saprobes, pathogens, and endophytes and shows a worldwide distribution (Phillips et al., 2013). Wijayawardene et al. (2022) accepted 22 genera in *Botryosphaeriaceae*. During our collecting programs of fungi inhabiting *M. grandiflora*, we collected three collections of *Botryosphaeriaceae* taxa. According to our knowledge, these are the first records of the above mentioned taxa reported from *M. grandiflora*.

***Botryosphaeria*** Ces. & De Not., Comm. Soc. crittog. Ital. 1(fasc. 4): 211 (1863)

Index Fungorum Registration Identifier IF 635

#### Notes

The genus *Botryosphaeria* was introduced by Cesati and De Notaris (1863) who did not designate the type. Barr (1972) proposed *B. dothidea* (Moug.:Fr.) Ces. & De Not. as the lectotype. Slippers et al. (2004) designated the neotype and epitype of *B. dothidea*. Phillips et al. (2013) comprehensively revisited the genus and accepted six species including *B*. *dothidea* while providing illustration and description for asexual morph.

**Figure 4:**
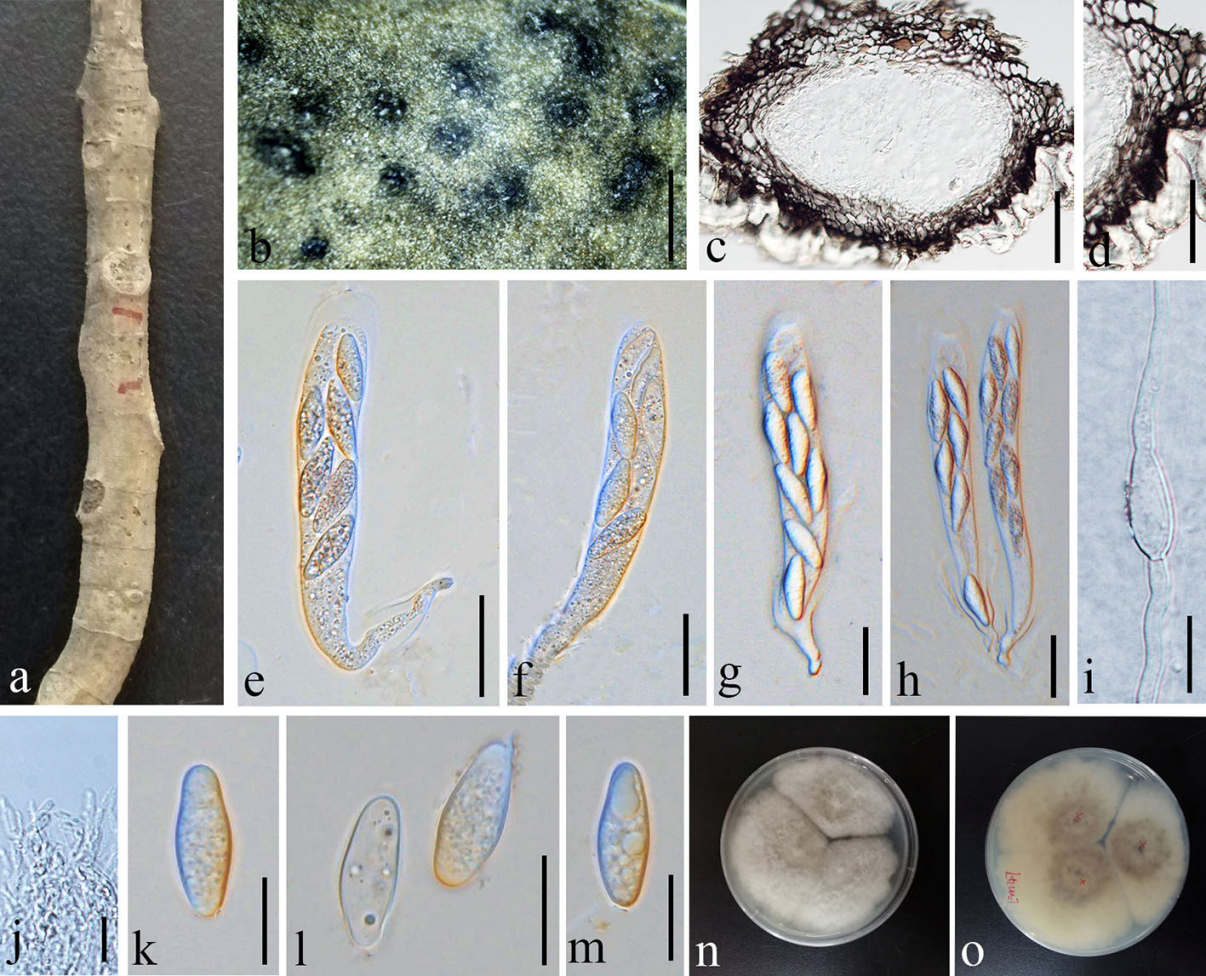
*Botryosphaeria dothidea* (GMB1387, new host record from China). **(a)** Host material. **(b)** Appearance of ascomata on the host. **(c)** Vertical section of ascoma. **(d)** Peridium. **(e–h)** Asci. **(i)** Germinating ascospore. **(j)** Pseudoparaphyses. **(k–m)** Ascospores. **(n)** Culture on PDA from above. **(o)** Culture on PDA from the bottom. Scale bars: **(b)** = 1,000, **(c)** = 50, **(d)** = 25, **(e–h)** =30, **(i,k–m)** = 15, and **(j)** =10 μm.

***Botryosphaeria dothidea*** (Moug.:Fr.) Ces. & De Not. 1863, (Figure 4)

Index Fungorum Registration Identifier IF 183247

*Saprobic* on dead (hanging) branches of *M. grandiflora*. Sexual morph: *Ascomata* 155–460 × 165–315 μm ($\bar{\text{x}}$ = 281.3 × 236.2 μm, *n* = 10), eustromatic, gregarious, black, uniloculate, with a thick pseudoparenchymatic wall composed of *textura angularis* or *textura globose* with the outer layers blackened and their cells more thickened, and erumpent at maturity. *Pseudoparaphyses* 2.5–3.5 μm ($\bar{\text{x}}$ = 2.9 μm, *n* = 20) wide, thin-walled, hyaline, aseptate, and constricted at the septa. *Asci* 65–110 × 15–20 μm ($\bar{\text{x}}$ = 84.4 × 17.7 μm, *n* = 20), clavate or cylindric-clavate, stipitate, bitunicate, ectotunica thin, endotunica rather thick, 3-layered, with a prominent apical chamber, 8-spored, and developing on a broad basal hymenial layer. *Ascospores* 20–25 × 7–9 μm ($\bar{\text{x}}$ = 23.8 × 7.6 μm, *n* = 20), irregularly biseriate in the ascus, hyaline, sometimes becoming pale brown with age, thin-walled, ovoid, fusoid, fusoid-ellipsoid, usually widest in the middle, straight or inequilateral, smooth, one-celled sometimes becoming 1–2 septate with age, contents smooth or granular, and may be guttulate. Asexual morph: undetermined.

#### Culture characteristics

Ascospores germinating on PDA within 24 h and germ tubes produced from both sides. Colonies growing fast on PDA, reaching 6 cm in 1 week at 28°C, effuse, velvety to hairy, circular, white in the first week, and brown to dark brown after 1 week from above and below.

#### Materials examined

China, Yunnan Province, Qujing Normal University, 25°52′36.75″N, 103°74′46.73″E, 1,853.8 m, on branch of *Magnolia grandiflora* L., 18 August 2021, Dong-Qin Dai and Mei-ling Zhu, Ling 47, GMB1387 (new host record), living culture, GMBCC1179.

#### Notes

Our new collection of *B. dothidea* from *M. grandiflora* morphologically resembles the type collection described in Phillips et al. (2013). In phylogenetic analyses, our collection (GMBCC1179) groups with *B. dothidea s. str*. (Figure 1). According to Deng (1963), Tai (1979), and Farr and Rossman (2022), *B. dothidea* has not been previously reported from *Magnolia* species in China. Zlatkovic et al. (2018) reported *B. dothidea* from *M. grandiflora* as a pathogenic species (a causal agent of stem and shoot dieback) from Serbia. However, we did not notice any disease symptoms in the host plant that we collected. Nevertheless, it is essential to collect more samples to confirm the impact of *B. dothidea* on *Magnolia* species, since it is an important ornamental plant in China. Here, we report *B. dothidea* from *M. grandiflora* in China for the first time.

***Diplodia*** Fr., In: Mont., Ann. Sci. Nat. Bot., sér. 2, 1: 302. 1834

Index Fungorum Registration Identifier IF 8047

#### Notes

Montagne (1834) introduced *Diplodia* with *D. mutila* (Fr.) Mont. as the type of species. Currently, 28 species are accepted in Wu et al. (2021). Members of *Diplodia* are distributed worldwide and occur as different life modes such as pathogens, saprobes, and endophytes (Phillips et al., 2013). Approximately, over 60 records of *Diplodia* species have been reported from China according to Xiao et al. (2021) and Farr and Rossman (2022). Nevertheless, *Diplodia* species have not been reported from *Magnolia* species in China. Here, we report *Diplodia mutila* and *D. seriata* from *M. grandiflora* for the first time in China. According to our knowledge, *Diplodia* species have not been reported from *M. grandiflora* so far.

**Figure 5:**
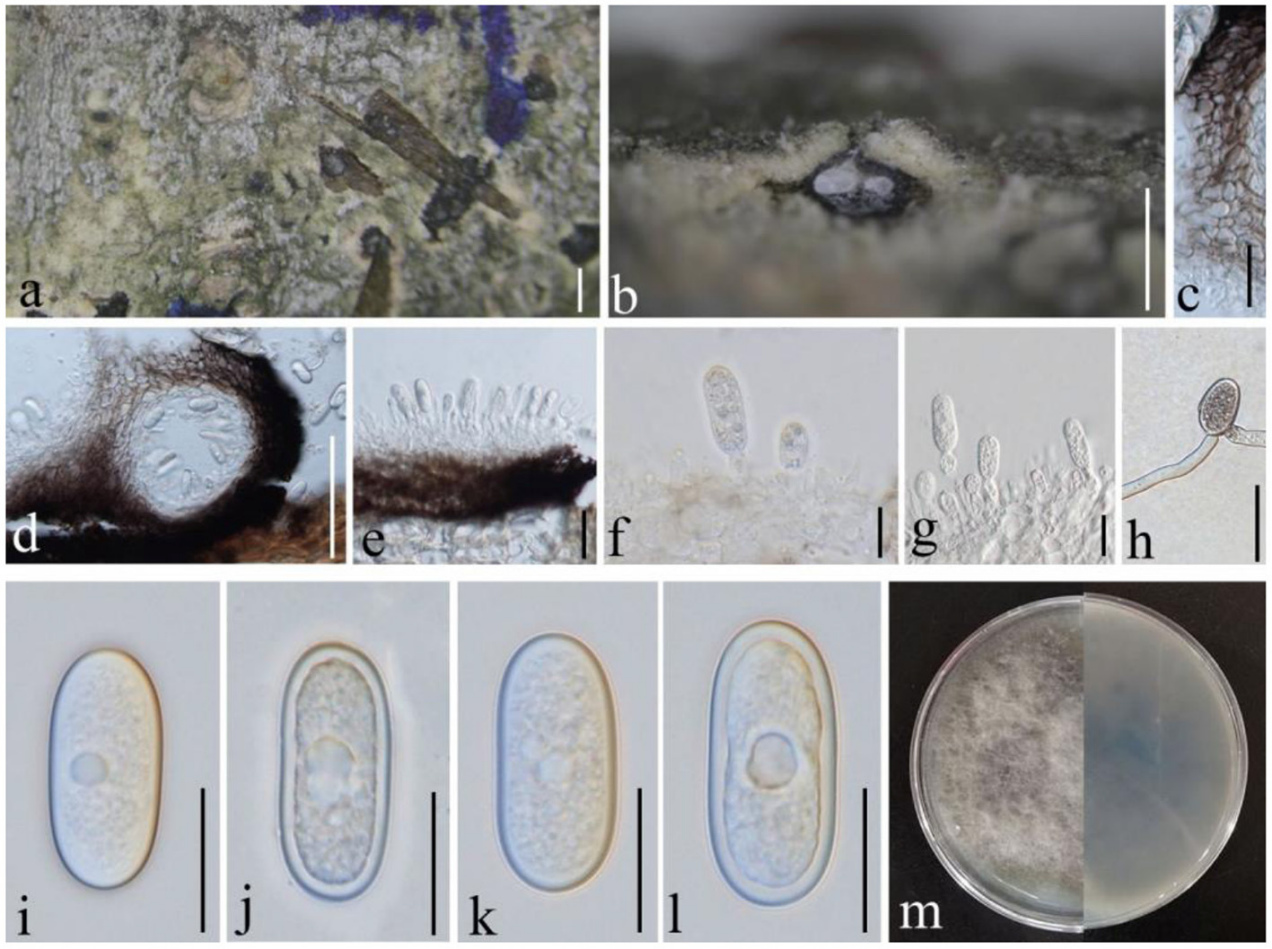
*Diplodia mutila* (GMB1381, a new host record). **(a)** Appearance of conidioma on the host. **(b,d)** Vertical sections of conidiomata. **(c)** Conidiomata walls. **(e–g)** Conidia attached to conidiog-enous cells. **(h)** Germinating conidium. **(i–l)** Conidia. **(m)** Cultures on PDA. Scale bars: **(a)** = 500, **(b)** = 200, **(c,g,h)** = 20, **(d)** = 100, **(e)** = 25, **(f)** = 10, and **(i–l)** = 15 μm.

***Diplodia mutila*** (Fr.) Mont., Annls Sci. Nat., Bot., sér. 2 1: 302 (1834), (Figure 5)

Index Fungorum Registration Identifier IF 201741

*Saprobic* on dead branches of *M. grandiflora*. *Conidiomata* 120–450 diam. × 160–400 μm high, solitary, immersed, partially erumpent at maturity, black, and globose. *Ostiole* central, circular, and papillate. *Conidiophores* reduced to conidiogenous cells. *Conidiogenous* cells 11–14 × 4–5 μm, holoblastic, discrete, cylindrical, hyaline, and smooth. *Conidia* 25–30 × 10–15 μm ($\bar{\text{x}}$ = 26.7 × 11.6 μm, *n* = 20), hyaline and aseptate at immature stage, smooth, thick-walled, oblong to ovoid, straight, both ends broadly rounded, and becoming pale brown at maturity.

#### Culture characteristics

Conidia germinating on PDA within 24 h and germ tubes produced from one side. Colonies growing fast on PDA, reaching 9 cm in 1 week at 28°C, effuse, velvety to hairy, circular, white in the first week, brown to dark brown from above after 1 week, and dark brown to lividity from below.

#### Material examined

China, Yunnan Province, Qujing Normal University, 25°52′36.75″N, 103°74′46.73″E, 1,853.8 m, on branch of *M. grandiflora* L., 7 September 2019, Dong-Qin Dai and Mei-ling Zhu, Ling 31, (GMB1381) (new host record), living culture GMBCC1173.

#### Notes

In morphology, our new collection closely resembles *Diplodia mutila* (Phillips et al., 2013) except for conidial width (10–15 vs. 13–14). However, in phylogenetic analyses, a new strain clusters with *D. mutila s. str*. with high statistical values (Supplementary Figure 2; 83% in ML analysis). Hence, we conclude that our collection is *D. mutila*, and it is the first report of this species from *M. grandiflora*.

**Figure 6:**
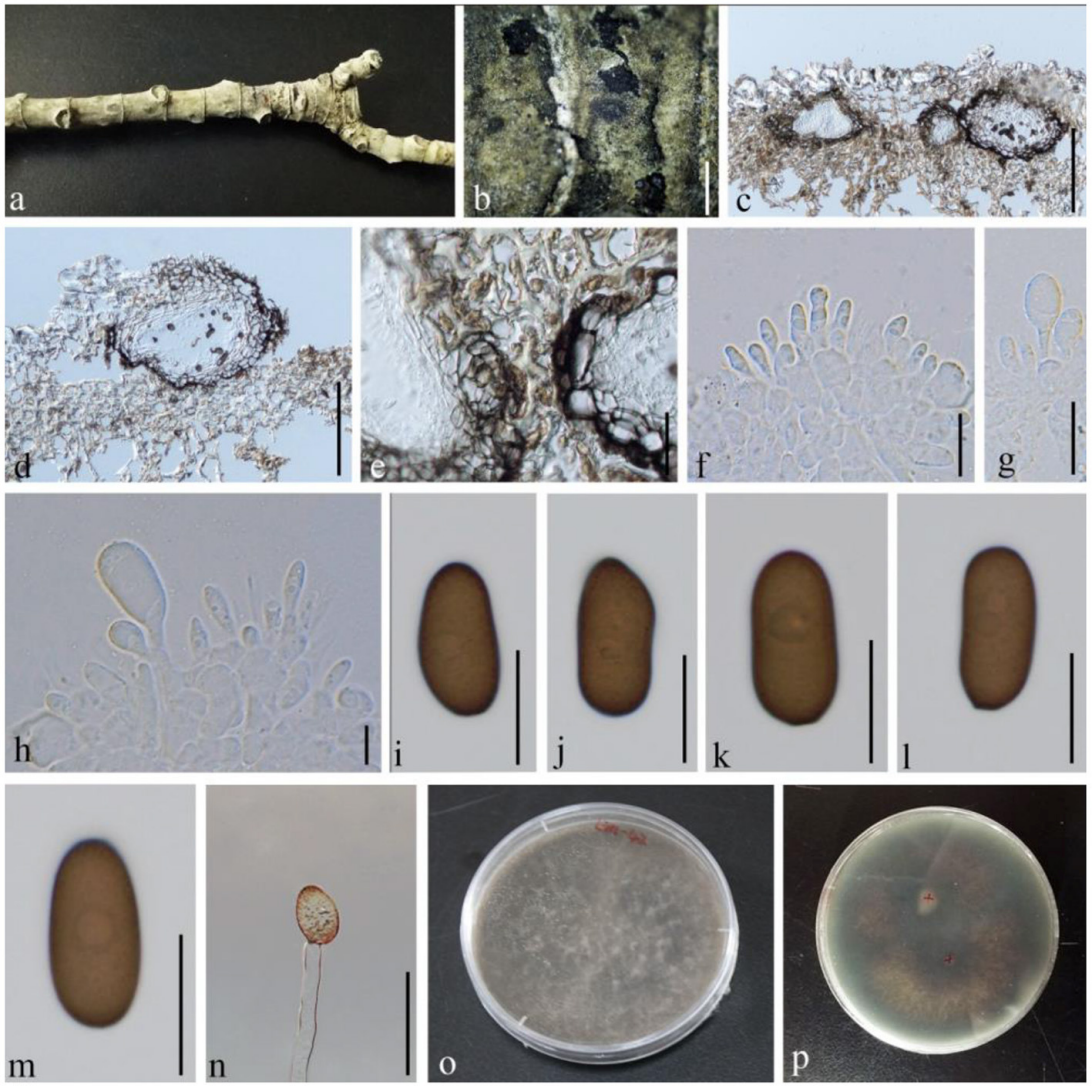
*Diplodia seriata* (GMB1383, a new host record). **(a)** *M. grandiflora* branch. **(b)** Appearance of conidiomata on the host. **(c,d)** Vertical sections of conidiomata. **(e)** Conidiomata walls. **(f–h)** Conidia attached to conidiogenous cells. **(i–m)** Mature conidia. **(n)** Germinating conidium. **(o,p)** Cultures on PDA. Scale bars: **(b)** = 1,000, **(c,d)** = 150, **(e)** = 30, and **(f–n)** = 15 μm.

***Diplodia seriata*** De Not., Mém. R. Accad. Sci. Torino, Ser. 2 7: 26 (1845), (Figure 6)

Index Fungorum Registration Identifier IF 201741

*Saprobic* on dead branches of *M. grandiflora*. *Conidiomata* 30–205 diam. × 23–65 μm high, solitary, immersed, partially erumpent at maturity, dark brown, and globose. *Ostiole* central, circular, and nonpapillate. *Conidiophores* reduced to conidiogenous cells. *Conidiogenous cells* 6–10 × 4–6 μm, holoblastic, discrete, cylindrical, hyaline, and smooth. *Conidia* 20–27 × 10–15 μm ($\bar{\text{x}}$ = 22.7 × 12.6 μm, *n* = 20), hyaline and aseptate at immature stage, smooth, thick-walled, oblong to ovoid, straight, both ends broadly rounded, and becoming dark brown at maturity.

#### Culture characteristics

Conidia germinating on PDA within 24 h and germ tubes produced from rear side. Colonies growing fast on PDA, reaching 9 cm in 1 week at 28°C, effuse, velvety to hairy, circular, white in the first week, brown to dark brown after 1 week from above, and black in the central and outermost circles with dark brown in the middle from below.

#### Material examined

China, Yunnan province, Qujing Normal University, 25°52′36.75“N, 103°74′46.73″E, 1,853.8 m, on branch of *M. grandiflora* L., 18 August 2020, Dong-Qin Dai and Mei-ling Zhu, Ling 42, (GMB1383) (a new host record), living culture GMBCC1175.

#### Notes

In conidial morphology, our new collection from *M. grandiflora* is morphologically similar to *Diplodia seriata* (Phillips et al., 2013). In our phylogenetic analyses, it was accommodated with *D. seriata s. str*. (Figure 1; Supplementary Figures 1, 2). Hence, we confirmed our new collection as *D. seriata*. According to Farr and Rossman (2022), *D. seriata* was not reported as from *M. grandiflora*. Hence, in here, we report *D. seriata* from *M. grandiflora* as a new host record in China.

***Pleosporales*** Luttr. ex M.E. Barr 1987

***Amorosiaceae*** Thambug. & K.D. Hyde, Fungal Diversity 74: 252 (2015)

Index Fungorum Registration Identifier IF 551277

#### Notes

Thambugala et al. (2015) introduced this family based on *Amorosia* Mantle & D. Hawksw. (type species: *Amorosia littoralis* Mantle & D. Hawksw.). At the same time, Thambugala et al. (2015) introduced *Angustimassarina* with *A. populi* Thambug. & K.D. Hyde as the type species. Currently, the family comprises five genera (Wijayawardene et al., 2022).

***Angustimassarina*** Thambug., Kaz. Tanaka & K.D. Hyde, Fungal Diversity 74: 253 (2015)

Index Fungorum Registration Identifier IF 551278

#### Notes

*Angustimassarina* was introduced by Thambugala et al. (2015) with three species and *A. populi* as the type species. Twelve records are listed in Index Fungorum (2022) while most of *Angustimassarina* species have been reported from Germany and Italy (Thambugala et al., 2015; Tibpromma et al., 2017; Hyde et al., 2019). Only one species, *A. populi*, was reported with both asexual and sexual morphs (Thambugala et al., 2015), while other species have been reported with only a sexual morph.

**Figure 7:**
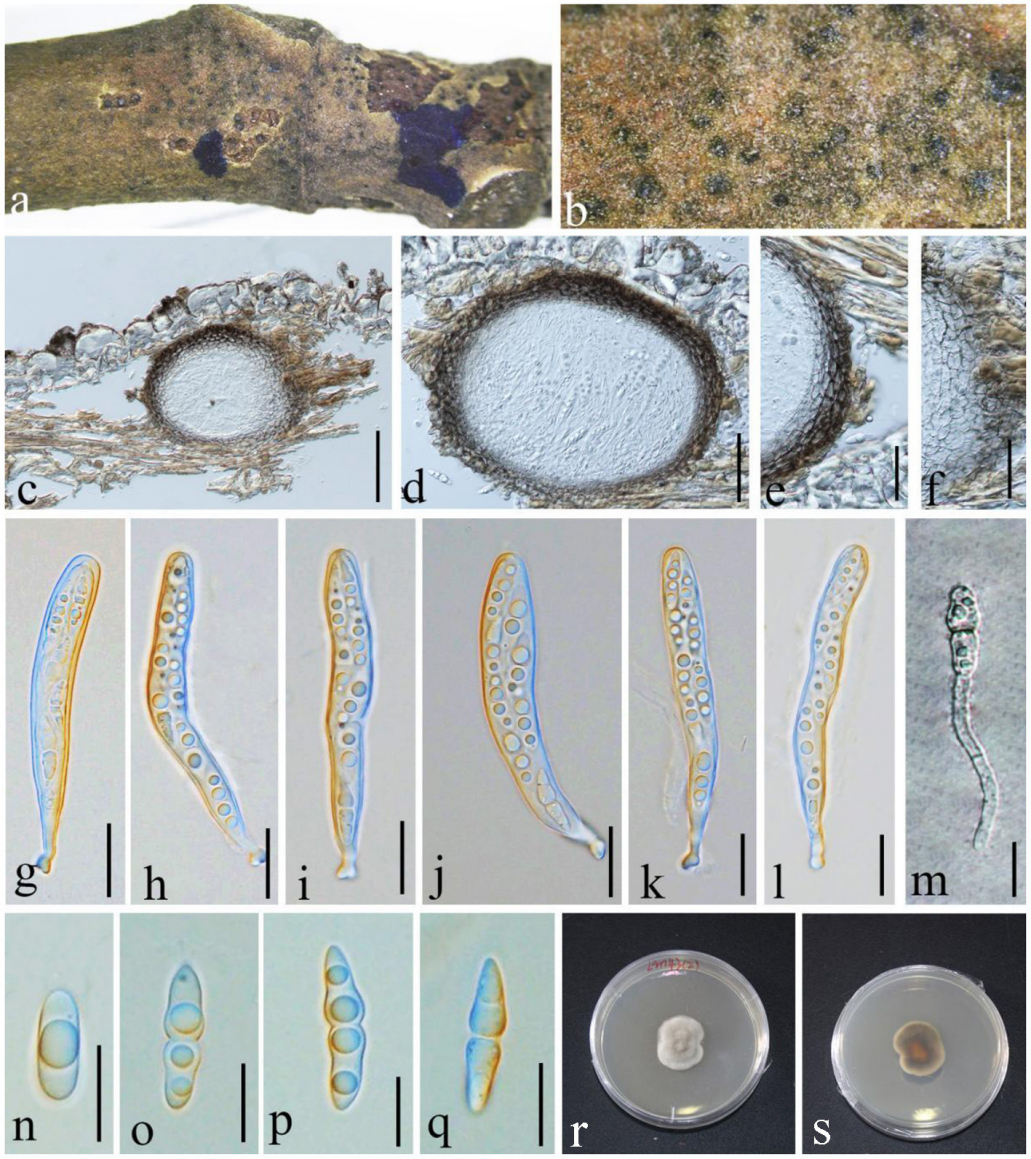
*Angustimassarina populi* (GMB1385, new host and country record). **(a,b)** Ascomata on a *M. grandiflora* branch. **(c,d)** Vertical sections of ascomata. **(e,f)** Peridium. **(g–l)** Asci. **(m)** Germinating ascospore. **(n–q)** Ascospores. **(r,s)** Cultures on PDA [**(r)** = from above and **(s)** = from bottom]. Scale bars: **(b)** = μm, **(c)** = 100, **(d)** = 50, **(e)** = 30, **(f)** = 25, **(g–l)** = 20, and **(m–q)** = 10 μm.

***Angustimassarina populi*** Thambug. & K.D. Hyde, Fungal Diversity: 10.1007/s13225-015-0348-3, [56] (2015), (Figure 7)

Index Fungorum Registration Identifier IF 551279

*Saprobic* on branch of *M. grandiflora*. Sexual morph: *Ascomata* 100–165 × 100–120 μm ($\bar{\text{x}}$ = 125.2 × 112.7 μm, *n* = 10), visible black dots, small dome-shaped on host surface, immersed, scattered, globose to sub-globose, uni-loculate, and without ostiole and papilla. *Peridium* 3–9 × 2–4.5 μm ($\bar{\text{x}}$ = 7.2 × 3.1 μm, *n* = 20), unequal thickness, thick-walled, composed of several layers of brown to dark brown, and arranged in *textura angularis*. *Hamathecium* composed of dense, 1.5–2 μm wide, filamentous, unbranched, septate, pseudoparaphyses, anastomosed between the asci, and embedded in hyaline gelatinous matrix. *Asci* 80–105 × 9–15 μm ($\bar{\text{x}}$ = 86.2 × 10.2 μm, *n* = 20), 8-spored, bitunicate, fissitunicate, cylindrical to cylindric-clavate, short with club-shaped pedicellate, and with well-developed ocular chamber. *Ascospores* 20–23 × 4–6 μm ($\bar{\text{x}}$ = 20.4 × 5.6 μm, *n* = 20), overlapping 1–2-seriate, fusiform, hyaline, 1 septate at the center, constricted at the septum, the upper cell larger than the lower cell, conical at the ends, guttulate, smooth-walled, and without mucilaginous sheath. Asexual morph: undetermined.

#### Culture characteristics

Ascospores germinating on PDA within 24 h and germ tubes produced from one side. Colonies grow on PDA at 28°C under normal light, reaching 2.5 cm diam. after 2 weeks, dense, irregular, umbonate, surface smooth, with edge entire, cottony, white to gray from above; white at the margin and dark brown at the center from below, and do not produce pigmentation in PDA.

#### Material examined

China, Yunnan Province, Qujing Normal University, 25°52′36.75“N, 103°74′46.73″E, 1,853.8 m, on branch of *M. grandiflora* L., 18 August 2021, Lin 43-2, (GMB1385; a new host and country record), living culture GMBCC1177.

#### Notes

In morphology, our new collection closely resembles *Angustimassarina populi* (holotype MFLU 14-0588). *Angustimassarina populi* was introduced by Thambugala et al. (2015), was collected from Italy on dead branches of *Populus* sp., and was characterized by erumpent, globose to subglobose ascomata with crest-like ostiole, cylindrical asci with fusiform ascospores, 1(−3)-septate, constricted at the central septum, and surrounded by a mucilaginous sheath; asexual morph is hyphomycetous. Based on our phylogenetic analyses of combined SSU, LSU, *tef1*, and ITS sequence data (Figure 2), our strain (GMBCC1177) clusters with the strains of *A. populi* at the basal clade of *A. arezzoensis* and *A. sylvatica*. Thus, we identify our fresh collection as *A. populi*. This is the first report of *A. populi* from China and on *M. grandiflora*. We also compared the morphology of the sexual morph of our new collection with *Angustimassarina* species that are morphologically and phylogenetically closely related (Table 3). However, the phylogenetic affinities of *Angustimassarina* species are not well-resolved (Figure 2). Therefore, to resolve the current status of *Angustimassarina*, further research is needed together with asexual morph and protein-coding genes.

**Table 3:** Morphological comparisons, location, and hosts of *Angustimassarina* species phylogenetically related to the new collection.

**Morphological characters**	**Species name**						
	***A. alni***	***A. arezzoensis***	***A. lonicerae***	***A. populi* (holotype)**	***A. populi* (new record)**	***A. premilcurensis***	***A. sylvatica***
Asexual morph	Undetermined	Undetermined	Undetermined	Hyphomycetous	Undetermined	Undetermined	Undetermined
**Sexual morph**							
Ascomata	160–250 × 130–200 μm	169–234 × 166–245 μm	193–203 × 170–220 μm	125–175 × 100–120 μm	100–165 × 100–120 μm	231–238 × 290–311 μm	180–260 × 150–200 μm
	Immersed to semi-immersed	Immersed to erumpent	Semi-immersed to erumpent	Immersed to semi-immersed becoming erumpent	Immersed	Immersed	Immersed to semi-immersed
	Globose to subglobose	Subglobose	Globose to subglobose	Globose to subglobose	Globose to subglobose	Globose to subglobose	Globose to subglobose
Papilla	No information	-	No information	+	–	–	–
Ostiole	+	+	+	+	–	+	+
Asci	71–89 × 8–10 μm	67–95 × 10–15 μm	55–81 × 9–13 μm	80–95 × 9.5–13 μm	80–105 × 9–15 μm	64–93 × 11–15 μm	95–110 × 8–12 μm
8-spored	+	+	+	+	+	+	+
	Cylindric-clavate	Broadly cylindrical to cylindric-clavate	Cylindrical	Cylindrical to cylindric-clavate	Cylindrical to cylindric-clavate	Cylindrical to cylindric- clavate	Cylindric-clavate
	Rounded at the apex with a minute ocular chamber	Rounded at the apex with a poorly develop ocular chamber	Rounded at the apex with a minute ocular chamber	Rounded at the apex with an ocular chamber	Well-developed ocular chamber.	Rounded at the apex with ocular chamber	Rounded at the apex
Ascospores	9–22 × 3–4 μm	19–21 × 5–6 μm	19–25 × 4–7 μm	19–22 × 3.2–5.5 μm	20–23 × 4–6 μm	19–23 × 4–7 μm	21–25 × 4–5 μm
Hyaline	+	+	+	Hyaline, becoming ocher brown at maturity	+	+	+
Fusiform	Fusiform to cylindrical or ellipsoidal-fusiform	+	+	Fusiform to cylindrical or ellipsoidal-fusiform	+	+	+
3-septate	+	+	1–3-septate	1–3-septate	1-septate at the center	1-septate	1-septate with 2 pseudosepta
Mucilaginous sheath	+	+	+	+	–	+	+
Countries/host	-Germany, *Alnus glutinosa*	Italy, *Salvia* sp.	Italy, *Lonicera* sp.	Italy, *Populus* sp.	China, *Magnolia grandiflora*	Italy, *Carpinus betulus*	Italy, *Fagus sylvatica*
References	Tibpromma et al. (2017)	Tibpromma et al. (2017)	Tibpromma et al. (2017)	Thambugala et al. (2015)	This study	Tibpromma et al. (2017)	Hyde et al. (2019)

+, present; –, absent.

***Parabambusicolaceae*** Kaz. Tanaka & K. Hiray. Stud. Mycol. 82: 115 (2015)

Index Fungorum Registration Identifier IF 811324

#### Notes

Tanaka et al. (2015) introduced this family to accommodate two genera, *viz*., *Aquastroma* and *Parabambusicola* (type genus). Members of the family have been mainly reported as saprobes. Currently, the family comprises nine genera (Wijayawardene et al., 2022).

***Lonicericola*** Phookamsak, Jayasiri & K.D. Hyde, Fungal Diversity 95(1): 39 (2019)

Index Fungorum Registration Identifier IF 556139

#### Notes

*Lonicericola* was introduced by Phookamsak et al. (2019) with *L. hyaloseptispora* Phookamsak et al. as the type of species. Later, Yasanthika et al. (2020) introduced the second species, *L. fuyuanensis* Yasanthika et al. Both have been introduced from Yunnan Province, China as saprobic species. Our new collection is morphologically resembling *Lonicericola s. str*. Multi-gene phylogenetic analyses and morphological characteristics confirmed that the new collections are new species in *Lonicericola*.

**Figure 8:**
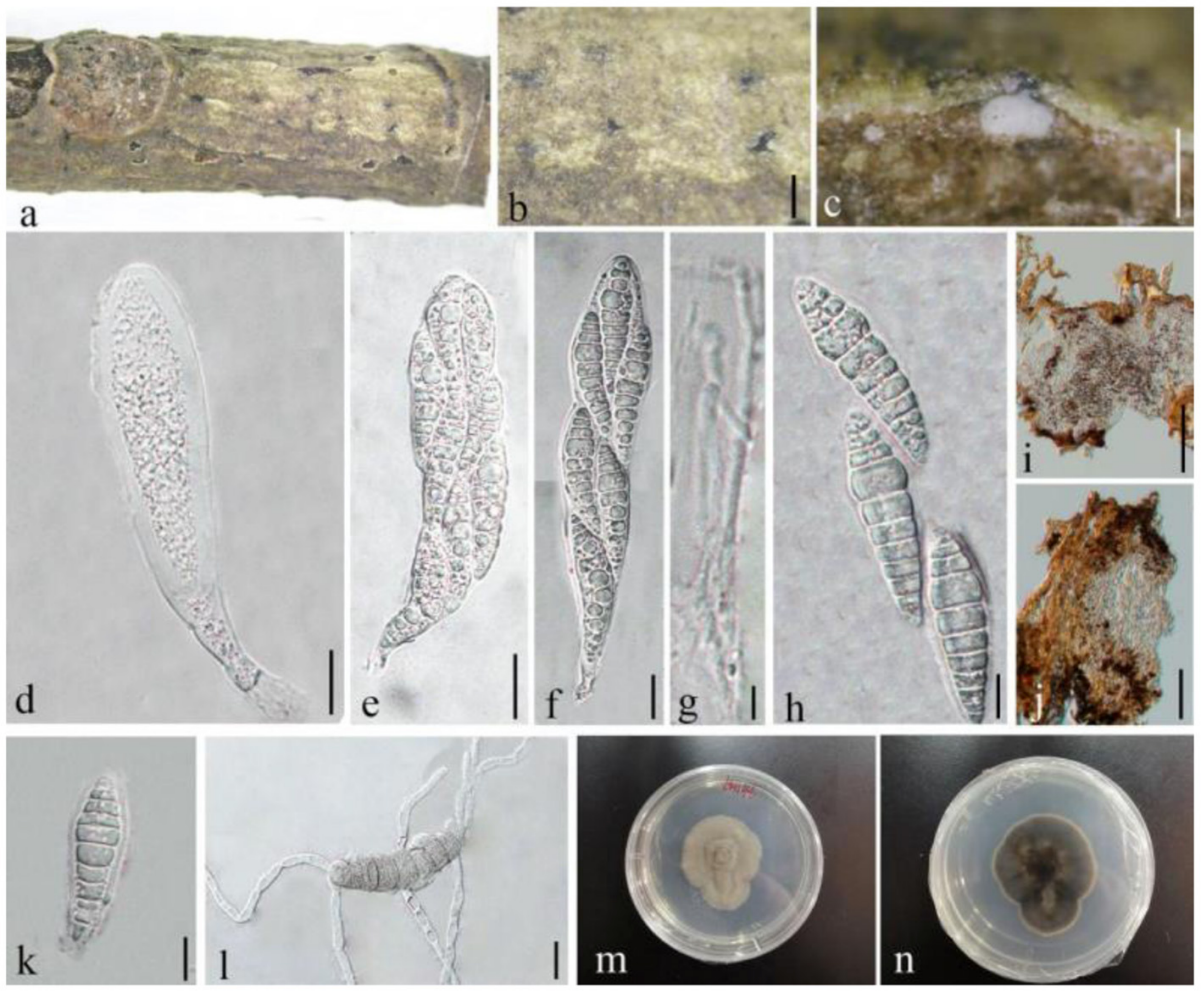
*Lonicericola qujingensis* (GMB1386, holotype). **(a)** Dead branch of *M. grandiflora*. **(b)** Immersed ascomata on host. **(c)** Vertical section of ascoma. **(d–f)** Asci. **(g)** Pseudoparaphyses. **(h,k)** Ascospores. **(i,j)** Peridium. **(l)** Germinated ascospore. **(m,n)** Seven-day-old colonies incubated at 28°C on PDA. Scale bars: **(b)** = 500, **(c,j)** = 50, **(d–f)** = 20, **(g,h,k,l)** = 10, and **(i)** = 100 μm.

***Lonicericola qujingensis*** D.Q. Dai, Wanas. & Wijayaw. sp. nov., (Figure 8)

Index Fungorum Registration Identifier IF 555252

Etymology: named after the locality from where it was collected, Qujing, Yunnan (China).

*Saprobic* dead branches of *M. grandiflora*, Sexual morph: *Ascomata* 250–300 × 100–160 μm ($\bar{\text{x}}$ = 284.2 × 129.7 μm, *n* = 10), black, scattered, solitary, immersed under host epidermis, slightly raised at maturity, globose to subglobose, uniloculate, glabrous, ostiolate, and papillate. *Ostiole* centrally located, oblong, with minute papilla, and filled with hyaline paraphyses. *Peridium* 10–20 μm wide, unequal thickness, composed of 2–3 layers, flattened to broad, brown to dark brown, composed of pseudoparenchymatous cells, and arranged in *textura angularis* to *textura prismatica*. *Hamathecium* composed of numerous, 1.5–3 μm wide, filamentous, and septate pseudoparaphyses. *Asci* 100–210 × 25–35 μm ($\bar{\text{x}}$ = 154.21 × 30.7 μm, *n* = 20), 8-spored, bitunicate, fissitunicate, broadly cylindrical to cylindric-clavate, subsessile to short pedicellate, with furcate to obtuse pedicel, and apically rounded. *Ascospores* 47–60 × 10–16 μm ($\bar{\text{x}}$ = 55.7 × 14.2 μm, *n* = 20), overlapping 2–3-seriate, hyaline, fusiform, 7–10-septate, constricted at the septa, smooth-walled, with small to large guttules, and surrounded by a mucilaginous sheath (14–18 μm diam.). Asexual morph: undetermined.

#### Culture characteristics

Ascospores germinating on PDA within 24 h and germ tubes produced from all sides. Colonies growing slowly on PDA, reaching 4 cm in diam. after 1 week at 28°C, dense, irregular, umbonate, surface smooth, with edge entire, cottony, white in the first week, white to gray from above; white at the margin and black in the center, and dark brown in the middle from below. Mycelium semi-immersed in PDA, with branches, septate, smooth-walled, and hyphae brown.

#### Material examined

China, Yunnan Province, Qujing, Qujing Normal University, 25°52′36.75″N, 103°74′46.73″E, 1,853.8 m, on dead branches of *M. grandiflora* L., 18 August 2020, Dong-Qin Dai and Mei-ling Zhu, Lin 46, (GMB1386, holotype), ex-type GMBCC1178; *Ibid*. 10 May 2021, Dong-Qin Dai and Ting-Ting Zhang, Lin 60, (GMB1046, paratype); ex-paratype GMBCC1037.

#### Notes

Currently, the genus comprises three species (including the new collection), and all species were reported from Yunnan, China. Interestingly, both *Lonicericola hyaloseptispora* and *L. fuyuanensis* have been reported from the same host family, i.e., *Caprifoliaceae*. Nevertheless, the new collection was made from decaying branches of *M. grandiflora* (*Magnoliaceae*). In our phylogenetic analyses (Figure 1), our new collection formed a distinct clade in *Lonicericola s. str*. with high bootstrap values (99% and 1 in ML and Bayesian analysis, respectively). This result is also supported by morphological characters (see the taxonomic key). Based on current data, we assume that *Lonicericola* species are restricted to subtropical regions in China but could be distributed in different host families.

The taxonomic key below can be used to distinguish the *Lonicericola* species based on ascospore and asci morphology.

Ascospores with only 3 septa …… *L. fuyuanensis*
Ascospores with more than 3 septa……2
Ascospores 37–49 × 8–12 μm, 8–9-septate…… *L*.hyaloseptispora^*^
Ascospores 47–60 × 10–16 μm, 7–10-septate…… *L. qujingensis*
^*^Phookamsak et al. (2019) did not provide ascospore dimensions; thus, we received them through personal communication with R. Phookamsak.

***Lentitheciaceae*** Y. Zhang ter, C.L. Schoch, J. Fourn., Crous & K.D. Hyde, Stud. Mycol. 64: 93 (2009)

Index Fungorum Registration Identifier IF 515470

#### Notes

Zhang et al. (2009) introduced this genus with *Lentithecium, Katumotoa*, and *Keissleriella*. Currently, *Lentitheciaceae* comprises 14 genera (Wijayawardene et al., 2022). Members of the family occur in both terrestrial and aquatic environments and are common as saprobes.

***Phragmocamarosporium*** Wijayaw., Yong Wang & K.D. Hyde, Index Fungorum 370: 1 (2018)

Index Fungorum Registration Identifier IF 555365

#### Notes

Wijayawardene et al. (2015) introduced this genus with two species, *P. hederae* Wijayaw. et al. (from *Hedera helix*, Germany) and *P. platani* (type species, from *Platanus* sp., Guizhou, China). Wanasinghe et al. (2018) introduced the third species, which was inhabitant on spines of *Rosa canina* from Great Britain. However, according to the Index Fungorum (2022), the genus was invalidly published in Wijayawardene et al. (2015); thus later, the genus and all the species have been validated in Index Fungorum (2022).

**Figure 9:**
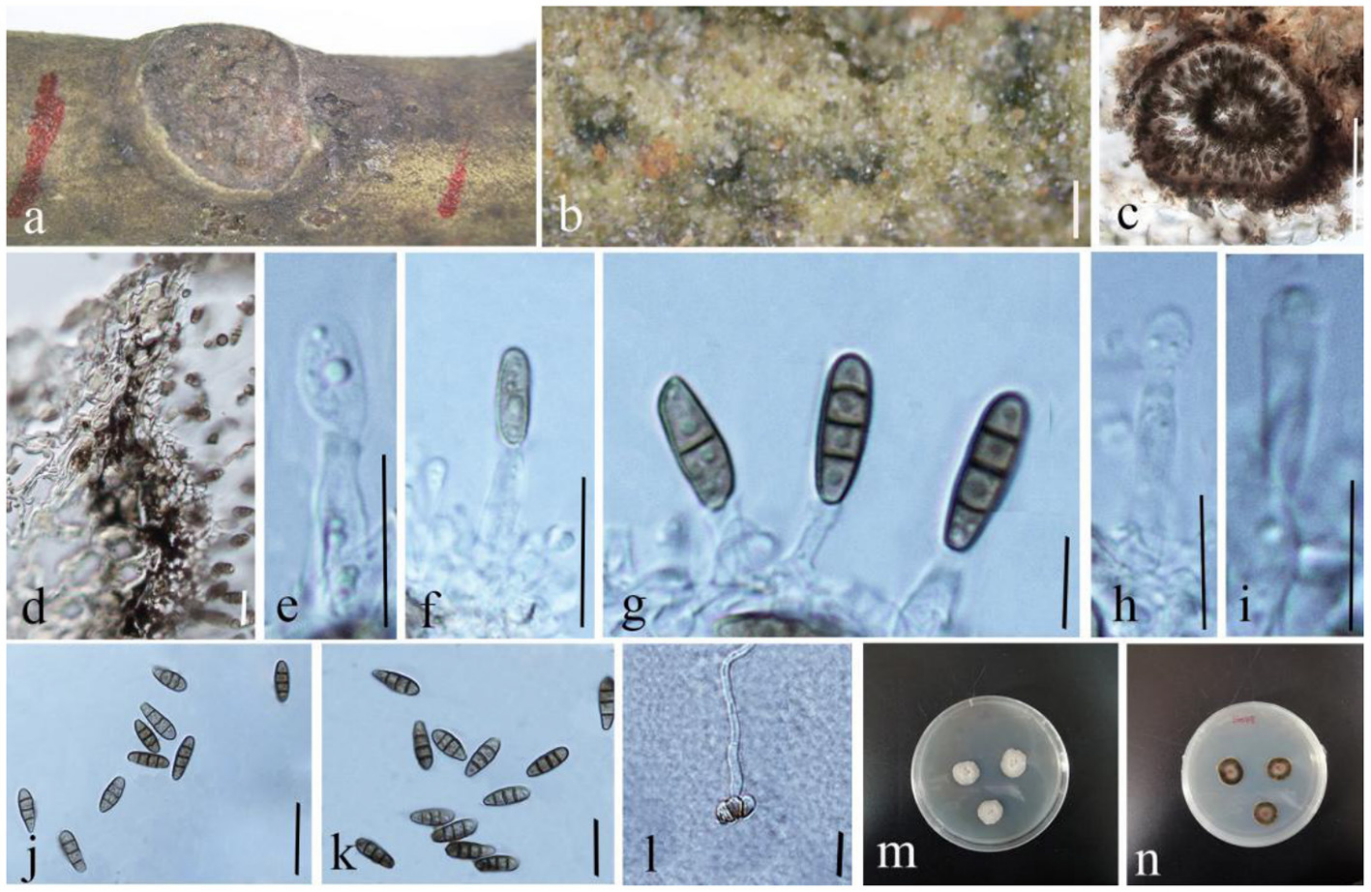
*Phragmocamarosporium magnoliae* (GMB1388, holotype). **(a)** Dead branch of *M. grandiflora*. **(b)** Appearance of conidiomata on host. **(c)** Vertical section of conidioma. **(d)** Pycnidium wall. **(e–i)** Developing and matured conidia attached to conidiogenous cells. **(j,k)** Conidia. **(l)** Germinated conidium. **(m,n)** Seven-day-old colonies incubated at 28°C on PDA. Scale bars: **(b)** = 100, **(c)** = 50, **(d)** = 20, **(e,g–i)** = 5, **(f,l)** = 15 μm, and **(j)** = 20 μm.

***Phragmocamarosporium magnoliae*** Wijayaw., D.Q. Dai & Wanas. sp. nov. (Figure 9)

Index Fungorum Registration Identifier IF 555250

Etymology: named after the host genus from which it was collected, *Magnolia*.

*Saprobic* on branches of *M. grandiflora*. Sexual morph: undetermined. Asexual morph: *Conidiomata* 100–145 μm high, 80–105 μm in diam. ($\bar{\text{x}}$ = 122.2 × 88.9 μm, *n* = 20), pycnidial, immersed, black, gregarious to solitary, unilocular, globose to subglobose, and with a centrally located papillate ostiole. *Pycnidial wall* with outer 3–4 layers of dark brown cells of *textura angularis*, with inner layer of thin hyaline cells. *Conidiophores* reduced to conidiogenous cells. *Conidiogenous cells* simple to simple branch at the base, smooth, long, 12–17 × 3–6 μm ($\bar{\text{x}}$ = 13.5 × 4.5 μm, *n* = 20), phialidic, and hyaline. *Conidia* 13–17 × 4–6 μm ($\bar{\text{x}}$ = 14.5 × 5.35 μm, *n* = 20), medium brown, clavate or ellipsoid to sub-cylindrical, with obtuse apex and truncate base, straight to curved, 3(−4)-transverse septate, guttulate or eguttulate, and constricted at the septa.

#### Culture characteristics

Conidia germinating on PDA within 24 h and germ tubes produced from the middle. Colonies growing slowly on PDA, reaching 2.5 cm in diam. after 1 week at 28°C, circular, zonate, uneven margin, cottony, white from above, with thin mycelium, dark brown at the margin, and yellowish-brown at the center from below. Mycelium semi-immersed in PDA, with branches, septate, smooth-walled, and hyphae brown.

#### Material examined

China, Yunnan Province, Qujing, Qujing Normal University, 25°52′36.75″N, 103°74′46.73″E, 1,853.8 m, on dead branches of *M. grandiflora* L., 18 August 2020, Dong-Qin Dai and Mei-ling Zhu, Lin 48, (GMB1388, holotype), ex-type GMBCC1180; *Ibid*. 10 May 2021, Dong-Qin Dai and Ting-Ting Zhang, Lin 68, (GMB1048, paratype); ex-paratype GMBCC1041.

#### Notes

In the phylogenetic analyses (Figure 3), *Phragmocamarosporium magnoliae* groups with *P. hederae* and *P. platani* (Figure 8), but in morphology they are different (Table 4). Besides, *P. hederae* and *P. platani* are lacking sequences of ITS and *tef1* loci. Species resolution of this subclade will be higher with more genes and more collections.

**Figure 10:**
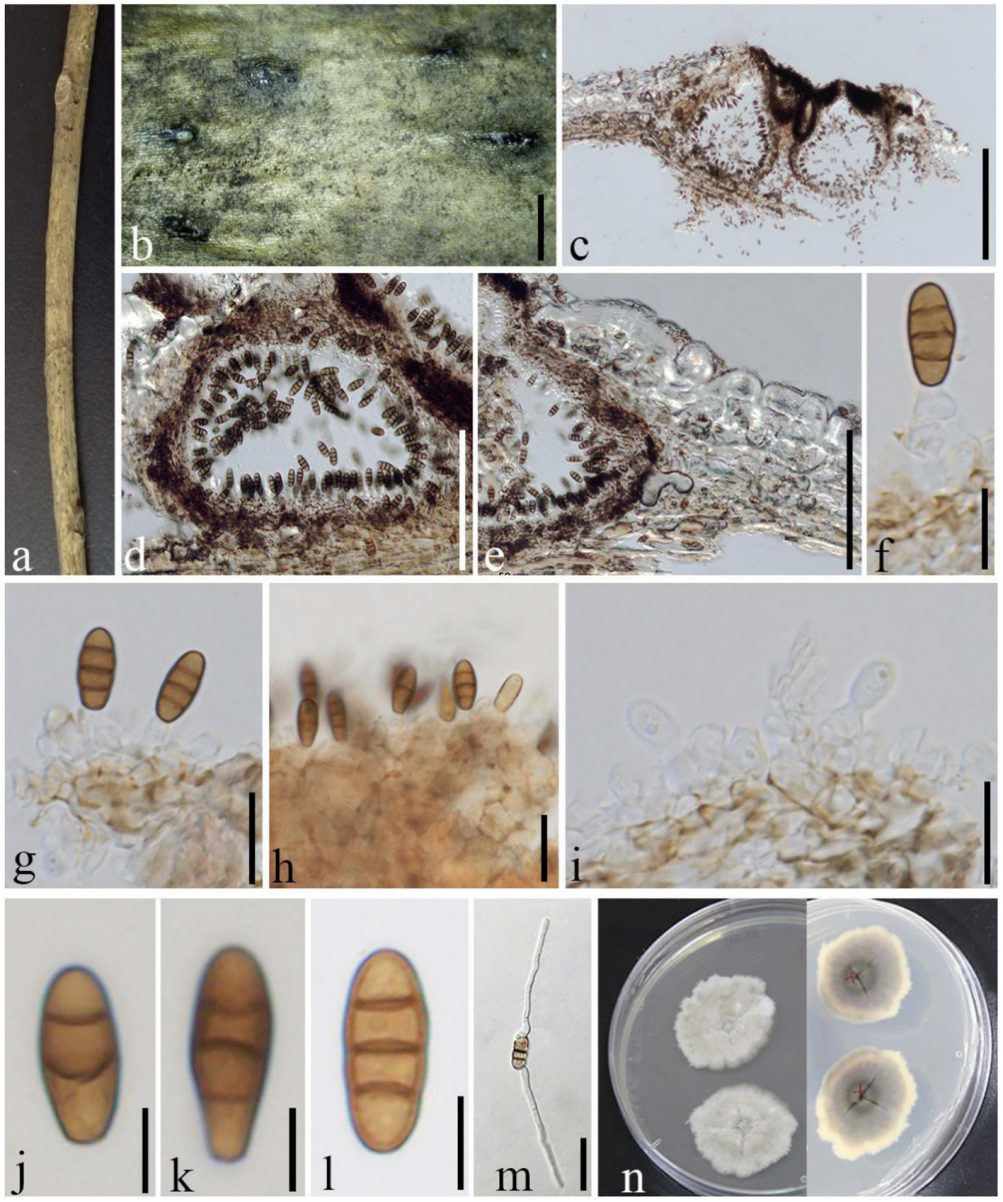
*Phragmocamarosporium qujingensis* (GMB1384, holotype). **(a)** Dead branch of *M. grandiflora*. **(b)** Appearance of ascomata on host. **(c,d)** Vertical sections of conidiomata. **(e)** Peridium. **(f–i)** Conidia attached to conidiogenous cells. **(j–l)** Conidia. **(m)** Germinated conidium. **(n)** Seven-day-old colonies incubated at 28°C on PDA. Scale bars: **(b)** = 1,000, **(c)** = 200, **(d)** = 100, **(e)** = 100, **(f,g,i)** = 10, **(h,m)** = 15, and **(j–l)** = 5 μm.

***Phragmocamarosporium qujingensis*** D.Q. Dai, Wanas. & Wijayaw. sp. nov., (Figure 10)

**Table 4:** Morphological comparison of *Phragmocamarosporium* species.

**Taxon and typification**	**Conidiomata**	**Conidiogenous cell**	**Conidia**	**Host/locality**	**References**
*P. hederae* (holotype)	80–110 μm high, 100–140 μm	8–10 × 1.5–2.5 μm	9–11 × 3–4.5 μm, 2-4-transverse septate	*Hedera helix*/ Germany	Wijayawardene et al. (2015)
*P. hederae* (reference collection)	130–170 μm high, 180–270 μm	3–5 × 2–5 μm	10–13, 3–4 μm, 3-transverse septate	*Cycas* (*Cycadaceae*)/Yunnan, China.	Phookamsak et al. (2019)
*P. platani* (holotype)	100–320 μm high, 150–300 μm	1.5–3 × 1.5–2.5 μm	12–13 × 5–7.5 μm, 3-4-transverse septate, rarely 1 longitudinal septa	*Platanus* sp./Guizhou, China	Wijayawardene et al. (2015)
*P. rosae* (holotype)	60–100 μm high, 120–200 μm	1–3 × 1–2.5 μm	8–10 × 3.5–4.5 μm, 3-transverse septate, 1 longitudinal septum	*Rosa* sp./The UK	Wanasinghe et al. (2018)
*P. qujingensis* (holotype)	100–300 μm high, 100–150 μm	6–11 × 2–5 μm	10–14 × 3–6 μm	*Magnolia grandiflora*/ Yunnan, China	This study
*P. magnoliae* (holotype)	100–145 μm high, 80–105 μm	12–17 × 3–6 μm	13–17 × 4–6 μm	*Magnolia grandiflora*/ Yunnan, China	This study

Index Fungorum Identifier IF 555251

Etymology: named after the locality from where it was collected, Qujing, Yunnan (China).

*Saprobic* on dead branches of *M. grandiflora*. Sexual morph: undetermined. Asexual morph: *Conidiomata* 100–300 μm high, 100–150 μm diam. ($\bar{\text{x}}$ = 142 × 122 μm, *n* = 10), pycnidial, immersed, semi immersed at maturity, black, gregarious to solitary, unilocular, globose to subglobose, and with a centrally located papillate ostiole. *Pycnidial wall* with outer 3–4 layers of dark brown cells of *textura angularis* and with inner layer of thin hyaline cells. *Conidiophores* reduced to conidiogenous cells. *Conidiogenous cells* 6–11 × 2–5 μm ($\bar{\text{x}}$ = 8.4 × 3.3 μm, *n* = 20), simple to simple branch at the base, smooth, long, phialidic, and hyaline. *Conidia* 10–14 × 3–6 μm ($\bar{\text{x}}$ = 11.5 × 4.4 μm, *n* = 30), medium brown, clavate or ellipsoid to subcylindrical, with obtuse apex and truncate base, straight to curved, 3(−4) transverse septate, guttulate or eguttulate, constricted at the septa.

#### Culture characteristics

Conidia germinating on PDA within 24 h and germ tubes produced from both sides. Colonies growing slowly on PDA, reaching 3 cm in 1 week at 28°C, dense, irregular, uneven margin, white in the first week, gray from above, light yellow at the margin and dark gray at the center, and with light gray in the middle from below.

#### Material examined

China, Yunnan Province, Qujing, Qujing Normal University, 25°52′36.75″N, 103°74′46.73″E, 1,853.8 m, on dead branches of *M. grandiflora* L., 18 August 2020, Dong-Qin Dai and Mei-ling Zhu, Lin 43-1, (GMB1384, holotype), ex-type GMBCC1176; *Ibid*. 10 June 2021, Dong-Qin Dai and Ting-Ting Zhang, Lin 101, (GMB1066, paratype); ex-paratype GMBCC1044.

#### Notes

*Phragmocamarosporium qujingensis* is morphologically and phylogenetically distinct from other species (Figure 3; Table 4).

***Longiostiolaceae*** Phukhams., Doilom, & K.D. Hyde, Fungal Diversity 102: 43 (2020)

***Shearia*** Petr., Annls mycol. 22(1/2): 180 (1924)

Index Fungorum Registration Identifier IF 9914

#### Notes

Petrak (1924) introduced this genus, which is typified by *Shearia magnoliae* (Shear) Petr. (Basionym: *Camarosporium magnoliae* Shear). However, Sutton (1980) regarded that *Stegonsporium formosum* Ell. & Ev. 1863 as the older name for this taxon. Thus, *S. formosa* (Ell. & Ev.) Petrak was regarded as the correct name for the type of species (Sutton, 1980). Wanasinghe et al. (2020) re-collected *Shearia formosa* from *Magnolia denudate* and *M. soulangeana* and designated the neotype. All collections were made from Kunming, Yunnan. In this study, we report *Shearia formosa* from *M. grandiflora* as a new host record from China (Wanasinghe et al., 2020; Farr and Rossman, 2022).

**Figure 11:**
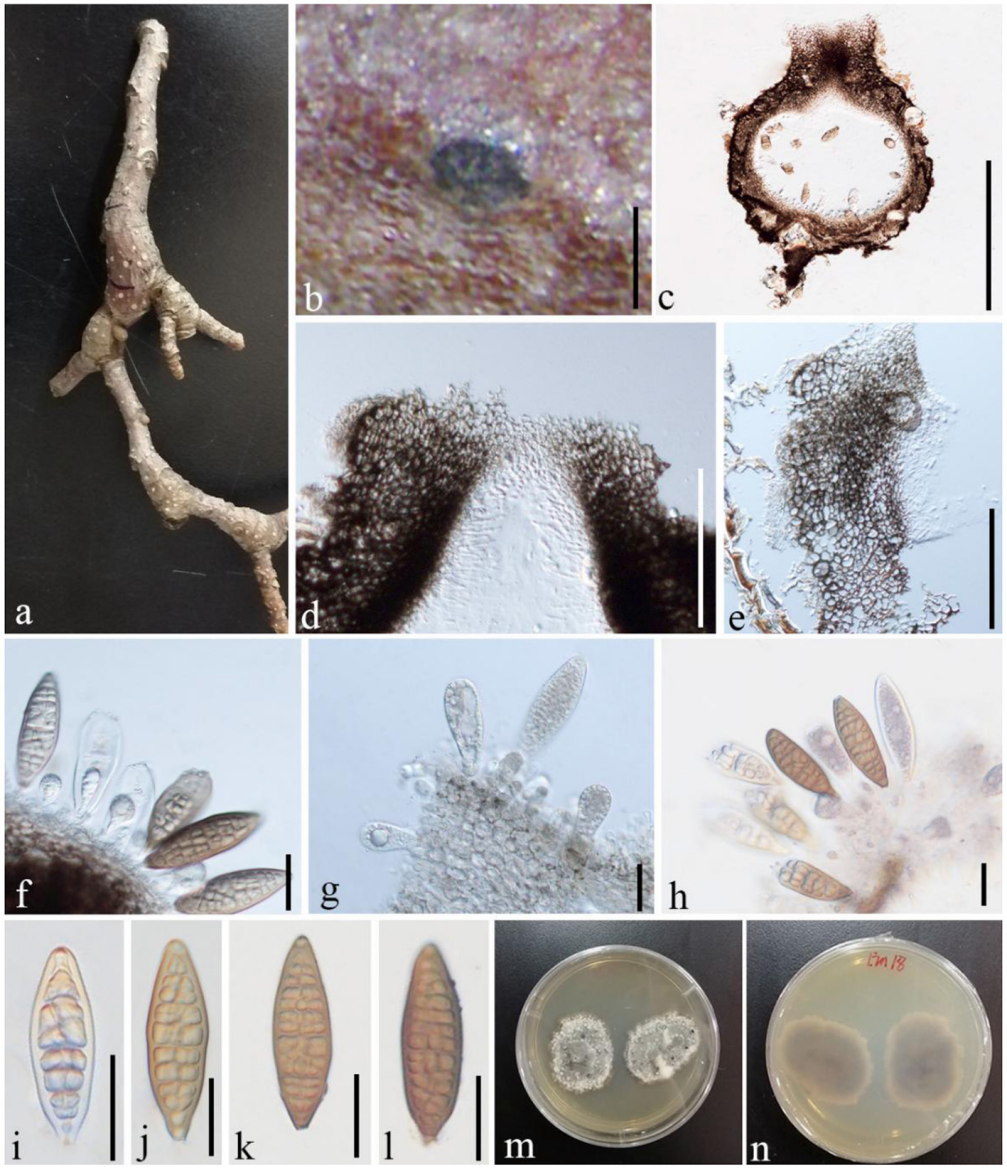
*S. formosa* (GMB1379, a new host record on *M. grandiflora* from China). **(a)** *M. grandiflora* branch. **(b)** Appearance of ascomata on host. **(c)** Vertical sections of conidioma. **(d)** Osti-ole opening. **(e)** Peridium. **(f–h)** Conidia and conidiogenous cells. **(i–l)** Conidia. **(m,n)** Seven-day-old colonies incubated at 28°C on PDA. Scale bars: **(b)** = 300, **(c)** = 430, **(d**) = 100, **(e)** = 55, **(f)** = 25, **(g)** = 20, **(h)** = 25, and **(i–l)** = 20 μm.

***Shearia formosa*** (Ellis and Everh.) Petr., Sydowia 15 (1–6): 216 (1962), (Figure 11)

Basionym: *Stegonsporium formosa* Ellis & Everh., Bull. Torrey bot. Club 10(7): 76 (1883)

Index Fungorum Registration Identifier IF 339263

*Saprobic* on dead twigs of *M. grandiflora*. Sexual morph: undetermined. Asexual morph: *Conidiomata* 500–870 μm high, 530–1,000 μm diam. ($\bar{\text{x}}$ = 635.5 × 763.3 μm, *n* = 20), pseudostromatic, solitary, immersed, globose, unilocular, dark brown, central, papillate ostiole, and circular. *Conidiomata wall* 15–35 μm wide at the base, 30–80 μm wide at the sides and ostiole region, outer layer composed of thick-walled, very dark brown occluded cells, and lateral and basal walls composed of dark brown cells of *textura angularis*. *Conidiophores* reduced to conidiogenous cells. *Conidiogenous cells* 7–14 × 6–8 μm ($\bar{\text{x}}$ = 11.2 × 7.3 μm, *n* = 10), holoblastic, annellidic, doliiform or cylindrical, discrete, inde-terminate, hyaline, and smooth-walled. *Conidia* 70–95 × 22–30 μm ($\bar{\text{x}}$ = 82 × 25 μm, *n* = 28), light brown to medium brown, fusiform, base truncate, apex obtuse, with several transverse and lateral distosepta, continuous, smooth and thick-walled, initially enveloped in a gelatinous sheath, depressed at the apex, and at maturity remaining as a basal lateral sheath.

#### Culture characteristics

Ascospores germinating on PDA within 24 h and germ tubes produced from both sides. *Colonies* growing slowly on PDA, reaching 3 cm in 1 week at 28°C, effuse, velvety to hairy, oval, uneven margin, white in the first week, white at the margin and gray at the center from above, and white at the margin and dark brown at the center from below.

#### Material examined

China, Yunnan Province, Qujing Normal University, 25°52′36.75″N, 103°74′46.73″E, 1,853.8 m, on branches of *M. grandiflora* L., 7 November 2019, Dong-Qin Dai and Mei-Ling Zhu, Lin 18, GMB1379 (new host record from China), culture GMBCC1172.

#### Notes

In conidial morphology and dimensions (in mean values), the new collection resembles the neotype of *Shearia formosa* (which was reported on *Magnolia denudate*) but is distinct from conidiomatal and conidiogenous cell characters (Wanasinghe et al., 2020) (Table 5). However, in phylogenetic analyses, the new collection accommodated in *Shearia s. str*. and clustered with MFLUCC 20–0019, the ex-neotype (Figure 1). Hence, we confirm our collection as *Shearia formosa*. *S. formosa* has previously been reported on *M. grandiflora* from the United States (Miller, 1990; Schubert, 1991; Farr and Rossman, 2022). However, according to our knowledge, *S. formosa* has not been reported on *M. grandiflora* from China. Hence, this is the first host record of *S. formosa* on *M. grandiflora* from China.

**Table 5:** Morphological comparison of new collection and neotype of *Shearia formosa*.

**Specimen**	**Conidiomata dimensions**	**Conidiogenous cells**	**Conidia dimensions**
*Shearia formosa* (neotype) on *Magnolia denudata* Wanasinghe et al. (2018)	500–700 μm high, 600–900 μm diam. ($\bar{\text{x}}$ = 636.4 × 795.1 μm, *n* = 10)	8–12 μm long, 5–8 μm wide ($\bar{\text{x}}$ = 9.9 × 6.4 μm, *n* = 20)	70–95 μm × 24–30 μm ($\bar{\text{x}}$ = 82.8 × 26.9 μm, *n* = 30)
New collection from *M. grandiflora* (this study)	500–870 μm high, 530–1,000 μm diam. ($\bar{\text{x}}$ = 635.5 × 763.3 μm, *n* = 20)	7–14 × 6–8 μm ($\bar{\text{x}}$ = 11.2 × 7.3 μm, *n* = 10)	70–95 × 22–30 μm ($\bar{\text{x}}$ = 82 × 25 μm, *n* = 28)

## Discussion

Tropical and subtropical regions are rich in biodiversity. Several studies concluded that some regions in Asia have not been properly studied; thus, a large number of fungal species are yet to be discovered (Hyde et al., 2018). In China, the southwestern region has higher biodiversity including higher floral, faunal, and microbial diversity (Xu et al., 2017). The Guizhou and Yunnan provinces are important in this region as a large number of research studies confirmed rich fungal diversity (Farr and Rossman, 2022).

In southwest China (i.e., Guizhou and Yunnan Provinces), *Magnolia* species are widely used in gardening (as an ornamental plant), horticulture, and Chinese traditional medicine. In this study, we focused on *M. grandiflora* in Qujing Normal University Garden, Qujing city, Yunnan province. We recognized that this species has been widely used in Qujing for gardening purposes. Thus, we selected the university garden as a preliminary collecting site to assess the fungal diversity of *M. grandiflora*.

*Botryosphaeriaceae* species are common in southwest China and other regions as well (e.g., Wijayawardene et al., 2016; Xiao et al., 2021). Our new collections of *Botryosphaeriaceae* taxa from *M. grandiflora* resided in *Botryosphaeria sensu stricto* and *Diplodia sensu stricto* (Figure 1). Among the taxa, one species is confirmed as *B. dothidea* while two other strains are confirmed as *D. mutila* and *D. seriata*. This is the first report of both *Diplodia* species on *M. grandiflora*. *Botryosphaeria dothidea* has been reported as a pathogen of broad range of hosts in China, including gardening plants (e.g., causal agent of trunk and extended up to branches of *Acer platanoides fide*; Wang et al., 2015) and agricultural crops (e.g., causal agent of apple ring rot of apple *fide*; Tang et al., 2012). According to Farr and Rossman (2022), *Botryosphaeria dothidea* has not been reported from *Magnolia* species from China; thus this is the first report. Nevertheless, *Botryosphaeria dothidea* was reported as a pathogen of *M. grandiflora* from Serbia (Zlatkovic et al., 2018). However, none of the new collections have been observed associated with any diseased symptoms such as cankers or leaf spots. Besides, in a recent genomic study by Yan et al. (2018), they predicted that some *Botryosphaeriaceae* species (e.g., *Lasiodiplodia theobromae*) could be opportunistic pathogens of woody plants with changes in the environment. Hence, it is essential to expand the sample number to confirm whether their life modes are adversely impacted by *M. grandiflora* populations in Qujing.

*Angustimassarina populi* was introduced as a saprobe of dead branches of *Populus* sp. from Italy (Thambugala et al., 2015). The genus *Angustimassarina* comprises twelve species epithets (Index Fungorum 2022) including *A. populi*, and all species have been reported from Europe. In this study, we reported *Angustimassarina populi*, which occurred on *M. grandiflora* from Qujing, Yunnan. This is the first report of a member of *Angustimassarina* reported outside Europe. Moreover, this is the first report of *Angustimassarina populi* from China and on *M. grandiflora* and, thus, the first country and host records, respectively. This collection confirms that *Angustimassarina* species could have a broader distribution and, apparently, are not host-specific. It is necessary to promote biogeographic studies of this type of genus, which was previously reported only in one geographic region but recently found in other countries. Based on this result, we predict that more novel species can be reported from China as *Angustimassarina* was originally reported in temperate countries, i.e., Italy.

The novel species of *Lonicericola, L. qujingensis* is the third member of the genus. Interestingly, all the species have been reported only from Yunnan Province, China. However, previous species (i.e., *L. hyaloseptispora* and *L. fuyuanensis*) have been reported from the host family *Caprifoliaceae*. Since the new collection was made from decaying branches of *M. grandiflora*, we predict that members of *Lonicericola* could occur in a broad range of host families. However, geographical distribution is not clear and thus needs further collections from other regions in Yunnan.

Currently, the genus *Phragmocamarosporium* comprises three species that were reported from Germany, The United Kingdom, and Guizhou Province, China. In this study, we introduce two more species of *Phragmocamarosporium* from *M. grandiflora viz*., *Phragmocamarosporium magnoliae* and *P. qujingensis*. *Phragmocamarosporium platani*, the type of species of *Phragmocamarosporium*, was reported from *Platanus* species, in Guizhou. Species resolution in the subclade in which *Phragmocamarosporium magnoliae, P. hedeare*, and *P. platani* are included is not clear as the latter species are lacking ITS and protein loci in the GenBank (Wijayawardene et al., 2015). Hence, here, we used morphological characteristics to differentiate the species as a supporting factor. Besides, we predict that the Guizhou-Yunnan region could be harboring more *Phragmocamarosporium* species. Moreover, the strain named KUMCC 18-0165 (of *Phragmocamarosporium hederae*) in the GenBank must represent a novel lineage in *Phragmocamarosporium s. str*. (Figures 1, 10; Table 4). Wanasinghe et al. (2020) reported *S. formosa* on *Magnolia denudate* from Yunnan, China. In this study, we report *S. formosa* on *M. grandiflora* for the first time.

Our findings suggest that it is essential to check for fungal diversity on extensively studied host genera that occur in biodiversity-rich regions. Hence, we suggest expanding future studies on extensively studied host genera that occur in Yunnan such as *Eucalyptus, Magnolia*, and *Quercus*. Besides, these host genera could be species-rich and thus could harbor different fungal taxa. Hence, precise host identification is also important in this type of broad future study. This is essential to reveal hidden fungal diversity in biodiversity hotspots.

## Data availability statement

The original contributions presented in the study are included in the article/Supplementary material, further inquiries can be directed to the corresponding authors.

## Author contributions

NW and D-QD designed the study, performed the morphological study, and wrote the manuscript. DW and ST performed the phylogenetic analyses. JK, H-HC, and D-QD reviewed and edited the manuscript. T-TZ, G-QZ, and M-LZ made the plates. L-SH performed the fungal DNA extraction. All authors approved the final version of the manuscript.

## Funding

The research was supported by the National Natural Science Foundation of China (Nos. NSFC 31860620, 31950410558, 31760013, and 32150410362), High-Level Talent Recruitment Plan of Yunnan Province (Young Talents Program and High-End Foreign Experts Program), CAS President's International Fellowship Initiative (Grant No: 2021FYB0005), and Postdoctoral Fund from Human Resources and Social Security Bureau of Yunnan Province, and it was partially supported by Chiang Mai University, Thailand.

## Conflict of interest

The authors declare that the research was conducted in the absence of any commercial or financial relationships that could be construed as a potential conflict of interest.

## Publisher's note

All claims expressed in this article are solely those of the authors and do not necessarily represent those of their affiliated organizations, or those of the publisher, the editors and the reviewers. Any product that may be evaluated in this article, or claim that may be made by its manufacturer, is not guaranteed or endorsed by the publisher.
