# Supplementary figures and images for: Fungi associated with dead branches of Magnolia grandiflora: A case study from Qujing, China
Source: Front Microbiol. 2022 Aug 4;13:954680. doi: 10.3389/fmicb.2022.954680 (PMC9386272; doi:10.3389/fmicb.2022.954680)

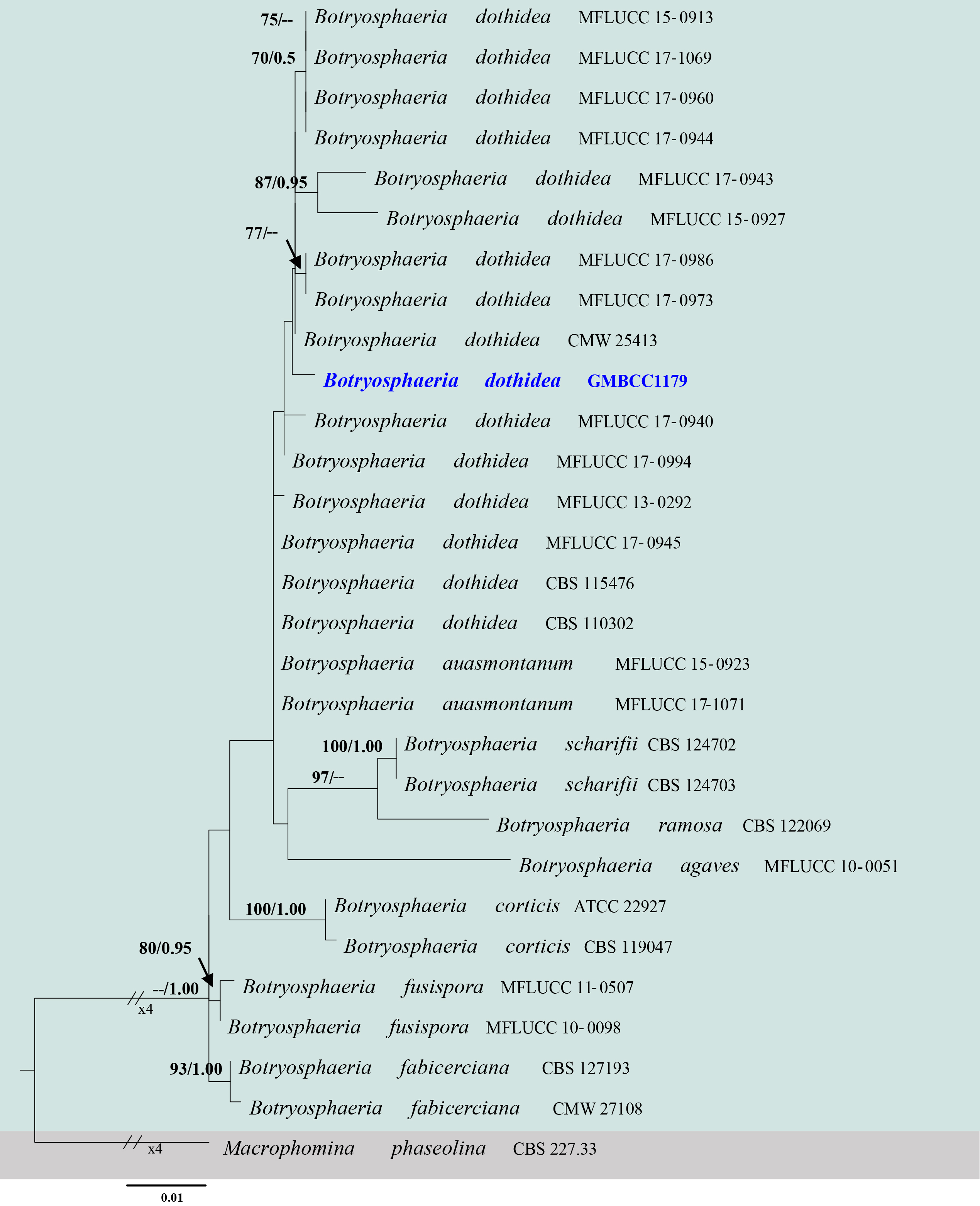

Supplement: Supplementary file 1 [file Image_1.JPEG]

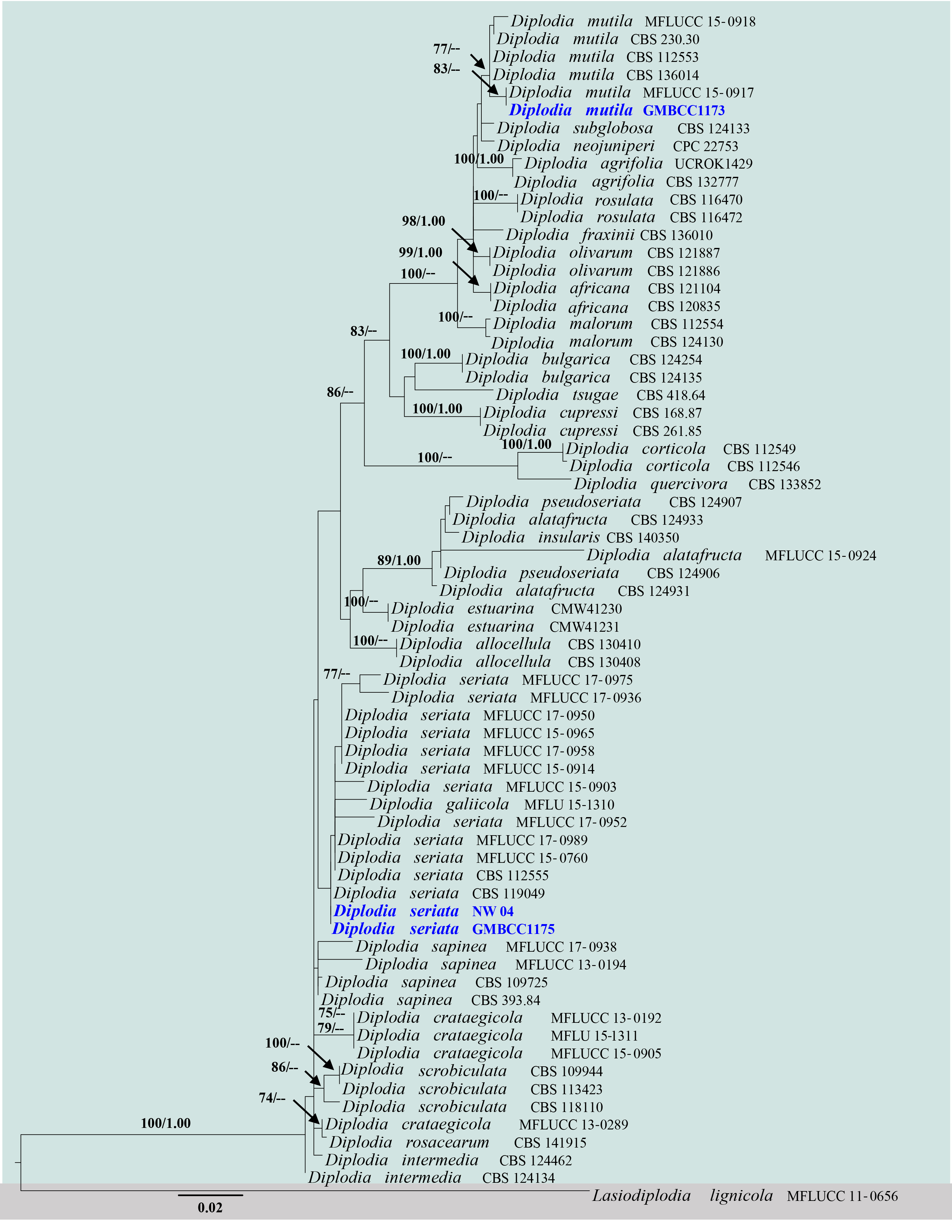

Supplement: Supplementary file 2 [file Image_2.JPEG]
